# Painted black: *Iguana melanoderma* (Reptilia, Squamata, Iguanidae) a new melanistic endemic species from Saba and Montserrat islands (Lesser Antilles)

**DOI:** 10.3897/zookeys.926.48679

**Published:** 2020-04-13

**Authors:** Michel Breuil, David Schikorski, Barbara Vuillaume, Ulrike Krauss, Matthew N. Morton, Elizabeth Corry, Nicolas Bech, Mišel Jelić, Frédéric Grandjean

**Affiliations:** 1 Muséum national d’Histoire naturelle, Laboratoire des Reptiles et Amphibiens, Bâtiment 30, 57, rue Cuvier, CP n° 30, 75231 Paris cedex 05, France Muséum national d’Histoire naturelle Paris France; 2 Laboratoire Labofarm-Genindexe, 4 rue Théodore Botrel, 22600 Loudéac, France Laboratoire Labofarm-Genindexe Loudéac France; 3 Maison du Soleil, Dauphin Road, La Borne, P O Box GM 1109, Saint Lucia Unaffliated Saint Lucia Saint Lucia; 4 Durrell Wildlife Conservation Trust, Les Augres Manor, Trinity, Jersey JE3 5BP, UK Durrell Wildlife Conservation Trust Trinity United Kingdom; 5 Laboratoire Écologie et Biologie des Interactions, équipe EES, UMR CNRS 6556, Université de Poitiers, 5 rue Albert Turpin, 86073 Poitiers Cedex 9, France Université de Poitiers Poitiers France; 6 Entomological Department, Varaždin City Museum, Šetalište Josipa Jurja Strossmayera 3, 42000 Varaždin, Croatia Varaždin City Museum Varaždin Croatia

**Keywords:** Conservation Biology, *
Iguana
*, Lesser Antilles, microsatellites, mtDNA, new endemic species, phylogeny

## Abstract

The Lesser Antilles, in the Eastern Caribbean, is inhabited by three *Iguana* species: the Lesser Antillean iguana*Iguana delicatissima*, which is endemic to the northernmost islands of the Lesser Antilles, the introduced common iguana from South America, *Iguana iguana iguana*, represented also by the two newly described endemic subspecies *Iguana iguana sanctaluciae* from Saint Lucia and *Iguana iguana insularis* from Saint Vincent and the Grenadines, and Grenada, and the introduced *Iguana rhinolopha* from Central America. Drawing on both morphological and genetic data, this paper describes the *Iguana* populations from Saba and Montserrat as a new species, *Iguana melanoderma*. This species is recognized on the basis of the following combination of characteristics: private microsatellite alleles, unique mitochondrial ND4 haplotypes, a distinctive black spot between the eye and tympanum, a dorsal carpet pattern on juveniles and young adults, a darkening of body coloration with aging (except for the anterior part of the snout), a black dewlap, pink on the jowl, the high number of large tubercular nape scales, fewer than ten medium sized–triangular dewlap spikes, high dorsal spikes, and lack of horns on the snout. This new melanistic taxon is threatened by unsustainable harvesting (including for the pet trade) and both competition and hybridization from escaped or released invasive alien iguanas (*I. iguana iguana* and *I. rhinolopha*) from South and Central America, respectively. The authors call for action to conserve *Iguana melanoderma* in Saba and Montserrat and for further research to investigate its relationship to other melanistic iguanas from the Virgin Islands and coastal islands of Venezuela.

## Introduction

**Figure 1.**
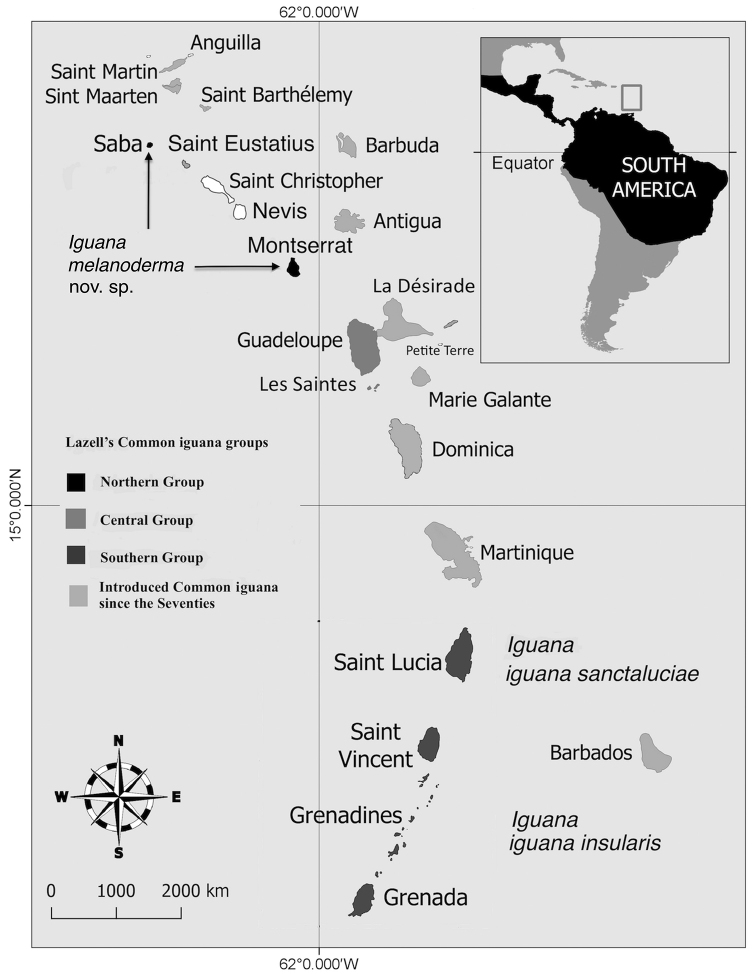
Geographical distribution of the three iguana groups identified by Lazell (1973) in the 1960s and new taxonomic proposition. In the 1960s, the invasive iguanas from South America (*Iguana iguana*) were only present in the îles des Saintes and Guadeloupe (Basse-Terre) and formed the Central Group. Now, alien iguanas are present and breed on every bank (van den Burg et al. 2018b). The southern group is now considered to support two subspecies *Iguana iguana insularis* and *Iguana iguana sanctaluciae* (Breuil et al. 2019). The northern group is considered here as a new species.

In the 1960s, Lazell (1973) studied the morphological variation of the Common Green Iguana (*Iguana iguana*) and identified three groups of this species in the Lesser Antilles (Fig. 1). The populations from Saba, Montserrat, and St. Croix were characterized by having large tubercular nape scales, highly developed dorsal crest spikes, a carpet pattern in 10–30 percent of the populations, an increasing incidence of melanistic individuals, and the absence of horns on the snout [1]. Populations of the Guadeloupian Archipelago and Les Saintes were distinguished by having very weak tubercular nape scales, and a high incidence of unpatterned and of grey individuals [2]. The southern Lesser Antillean populations, from Saint Lucia, Saint Vincent, the Grenadines, and Grenada were in turn characterized by very weakly tubercular nape scales, grey or green individuals with dorsolateral bands and highly developed, hornlike, median snout scales [3]. Lazell (1973) thought that geographic variation was clinal and concluded that the Common Iguana in Les Saintes and Guadeloupe was autochthonous rather than introduced.

Breuil (2002, 2013, 2016), Breuil et al. (2010), and Vuillaume et al. (2015) demonstrated that the Common Green Iguana present in Guadeloupe came from French Guiana and that this species displaced the Lesser Antilles iguanas (*Iguana delicatissima*) from Les Saintes and Grande-Terre through competition and hybridization. The same phenomenon is underway in Basse-Terre and St. Barthélemy leading to the further decline of *Iguana delicatissima* (Vuillaume et al. 2015; Breuil 2013, 2016). Since these recent studies, hybridization between these two species is known to have occurred in Martinique, La Désirade (MB, unpublished results), and St. Eustatius (Van den Burg et al. 2018a; 2018b). Thus, a natural cline concerning, for example, the size of the tubercular nape scales as advocated by Lazell (1973), cannot exist with only two groups of iguanas naturally present.

Morphological studies of the St. Lucia Iguana, belonging to the third group of Lazell (1973), indicated this iguana population is very different from the horned iguana population from Central America (Breuil 2013, 2016). Breuil et al. (2019) showed with genetic (microsatellites, mtDNA) and morphological studies that this third group comprises two subspecies, *Iguana iguana sanctaluciae*, endemic to Saint Lucia and *I. iguana insularis*, endemic to the Grenadine Bank.

In Saba, the local iguana, the Saban Black Iguana is considered as a flagship species. In Montserrat, the local melanistic iguana does not receive special attention due to its putative exotic origin. The two flagship herp species for conservation on the island are the mountain chicken (*Leptodactylus fallax*) and the Galliwasp (*Diploglossus montiserrati*).

Melanistic iguanas phenotypically close to those of Saba-Montserrat also occur in the northern islands (St. Croix, St. Thomas: USA Virgin Islands, USVI) and also on the island of Margarita and other coastal islands such as Los Roques and La Banquilla (Venezuela) (Lazell 1973; van Buurt 2005). Thus, further detailed investigations are required to establish the relationships among all these melanistic iguanas. It is noteworthy that Stephen et al. (2013) used in their phylogeny a unique individual from Venezuela that was captured on the mainland (Cumana) near Margarita Island and that clustered with melanistic iguanas from Saba and Montserrat.

The extension of the range of the Common Green Iguana from Central America, considered by Breuil et al. (2019) as a species in its own right (*Iguana rhinolopha*), to Saint Maarten (Vuillaume et al. 2015) increases the probability that this invasive lineage will arrive on the Dutch island of Saba (Yokoyama 2012). Morphological studies (Breuil 2013, 2016) combined with genetic studies (Vuillaume et al. 2015) have shown that the Saba population has unique characteristics. Stephen et al. (2013) found that Montserrat and Saba iguanas share the same ND4 haplotype. Montserrat conservationists also need to be able to differentiate between endemic melanistic iguanas and potential invasive Common Iguanas from Central and South American lineages that are likely to invade other islands (Breuil 2013, 2016; Falcón et al. 2013; Van den Burg et al. 2018b). With the increase in trade and shipping in the Caribbean region and post-hurricane restoration activities, it is very likely that there will be new opportunities for invasive iguanas to colonize new islands inhabited by endemic lineages.

The paper aims to describe the common melanistic iguanas from the islands of Saba and Montserrat as a new taxon and to establish its relationships with other Common Green Iguanas. An outcome of this study will be the enabling of conservationists to accurately differentiate this endemic lineage from invasive iguanas and investigate its ecology and biology population on these two very small islands that are subject to a range of environmental disturbances including hurricanes, earthquakes and volcanic eruptions.

## Materials and methods

Morphological, molecular (i.e., mitochondrial and microsatellites markers) and biological data were used to compare the iguanas of Saba and Montserrat with *Iguana iguana* (South America), *Iguana rhinolopha* (Central America), and the two new subspecies *I. iguana insularis* and *I. iguana sanctaluciae* from southern Lesser Antilles (Breuil et al. 2019).

### Morphological analyses

The morphological traits used to identify iguanas are described in Breuil (2013; 2016). Most consist of meristic and qualitative characteristics recorded on wild iguanas in the field on the islands of Saba and Montserrat, which are easily recorded from digital photographs taken by the authors. We also examined specimens at the Museum of Comparative Zoology (MCZ) at Harvard University, USA, based on photographs taken by Joseph Martinez and Corentin Bochaton (Muséum national Histoire naturelle, Paris).

We also reviewed photographs of *Iguana* found on the Internet using the Google Images search engine for the islands of St. Croix, St. Thomas (US Virgin Islands), for the coastal islets of Venezuela and for the vicinity of Cumana (Venezuela), regions known to be inhabited by melanistic iguanas, in relation to published data. Leighton et al. (2016) advocated the use of Internet images for taxonomic research to study spatial patterns in phenotypic traits that are objective, binary and easy to score, regardless of the camera angle. We used only photographs that were georeferenced to allow valid comparisons with the morphological characteristics of this new taxon (Breuil et al. 2019).

### Molecular analyses

Collection and preparation of genetic material

Genomic DNA was isolated from 44 individuals from tissue, shed skin and/or blood samples using the QIAamp DNA Mini Kit (QIAGEN, Deutschland) and following the manufacturer’s recommendations (Table 1).

**Table 1.** Sampling and haplotype information. The effectives in the locality column correspond to the number of individuals studied for the microsatellites and the mitochondrial ND4 gene. The number in the ND4 column is the length of the sequence used in the analysis. SabaNP, Saba National Park. SBHNR, Saint Barthélemy Natural Reserve. DWCT, Durrell Wildlife Conservation Trust.

Locality/Status	Microsatellites	ND4	Collectors	GenBank
**Saba (*N* = 7/*N* = 6)**	SABA01	SABA01 (888)	SabaNP/SBHRN	MN590163
	SABA02	SABA02 (890)	SabaNP/SBHRN	MN590164
	SABA03	SABA03 (739)	SabaNP/SBHRN	
	SABA04	SABA04 (892)	SabaNP/SBHRN	MN590165
	SABA05	SABA05 (892)	SabaNP/SBHRN	MN590166
	SABA06	SABA06 (892)	SabaNP/SBHRN	MN590167
	SABA07	SABA07 (891)	SabaNP/SBHRN	MN590168
**Montserrat (*N* = 12/*N* = 4)**	IGU86		E. Corry DWCT	
	IGU87		E. Corry DWCT	
	IGU88	IGU88 (713)	E. Corry DWCT	MN590169
	IGU89	IGU89(711)	E. Corry DWCT	MN590170
	IGU90		E. Corry DWCT	
	IGU91		E. Corry DWCT	
	IGU92	IGU92(713)	E. Corry DWCT	MN590171
	IGU93	IGU93(713)	E. Corry DWCT	MN590172
	IGU94		E. Corry DWCT	
	IGU95		E. Corry DWCT	
	IGU96		E. Corry DWCT	
	IGU99		E. Corry DWCT	
**St. Lucia /End (*N* 13/*N* = 2)**	IGU58	IGU63(779)	St. Lucia authors	MK687397
	IGU59		St. Lucia authors	
	IGU60		St. Lucia authors	
	IGU61		St. Lucia authors	
	IGU62		St. Lucia authors	
	IGU63	IGU63(737)	St. Lucia authors	MK687398
	IGU64		St. Lucia authors	
	IGU67		St. Lucia authors	
	IGU68		St. Lucia authors	
	IGU69		St. Lucia authors	
	IGU70		St. Lucia authors	
	IGU71		St. Lucia authors	
	IGU72		St. Lucia authors	
**Grenadines/End (*N* = 4/*N* = 4)**	IGU73		J. Daltry/G. Gaymes	MK787400
	IGU75		J. Daltry/G. Gaymes	MK787401
	IGU76		J. Daltry/G. Gaymes	MK787403
	IGU77		J. Daltry/G. Gaymes	MK787404
**Guiana/auto (*N* = 7/*N* = 4)**	IGU78	IGU78 (702)	F. Catzefis (CNRS)	MK687405
	IGU79	IGU79 (593)	F. Catzefis (CNRS)	MK687406
	IGU80		F. Catzefis (CNRS)	
	IGU81		B. de Thoisy (Pasteur)	
	IGU82	IGU82 (712)	B. de Thoisy (Pasteur)	MK687407
	IGU83		B. de Thoisy (Pasteur)	
	IGU84	IGU84 (697)	B. de Thoisy (Pasteur)	MK687408
	IGU85		B. de Thoisy (Pasteur)	

### Mitochondrial DNA (ND4) and phylogenetic analysis

Fragments of the ND4 mitochondrial locus, encompassing the 3' end of the NADH dehydrogenase subunit 4 gene (ND4) and the tRNA genes histidine, serine and leucine (partial 5' end), were PCR-amplified using the primer pair and protocols of Malone et al. (2000). Sequence chromatograms were analyzed in SEQUENCHER (v5.3; Gene Codes Corp., Ann Arbor). The resulting ND4 sequences were aligned with GenBank sequences of iguanas from previous studies (Malone et al. 2000; Malone and Davis 2004; Stephen et al. 2013; Breuil et al. 2019). Sequence alignment was calculated using MAFFT (v7.18) (Katoh et al. 2005). For the ND4 analysis, the molecular data set included 21 iguanas (Table 1) from insular and continental origins.

The best nucleotide substitution model was chosen using JModelTest 2 (Darriba et al. 2012) under the Bayesian information criterion (BIC). Hierarchical relationships between individual ND4 genotypes/haplotypes were analysed using Maximum Likelihood (ML) and Maximum Parsimony (MP) tree reconstructions, and Median-Joining (MJ) haplotype networks. The evolutionary history was inferred using the ML method based on the Hasegawa-Kishino-Yano model (Hasegawa et al. 1985). The initial trees for the heuristic search were obtained automatically by applying the Neighbor-Joining and BioNJ algorithms to a matrix of paired distances estimated using the Maximum Composite Likelihood (MCL) approach, then selecting the topology with the highest log likelihood value. A discrete Gamma distribution was used to model evolutionary rate differences between sites [5 categories (+G, parameter = 0.3079)]. The tree was drawn to scale, with branch lengths measured as the number of substitutions per site. The MP tree reconstruction was obtained using the Subtree-Pruning-Regrafting (SPR) algorithm (Nei and Kumar 2000) with a search level 1, in which the initial trees were obtained by random addition of sequences (10 replicates). Node supports for tree topologies obtained by ML and MP methods were calculated as percentages of replicate trees in which the associated taxa had clustered in the bootstrap test (1000 replicates). Node supports were indicated with bootstrap support (BS ≥ 70). ML an MP analyses were conducted in MEGA7 (Kumar et al. 2016). The MJ haplotype network (Bandelt et al. 1999) was constructed using PopART (Population Analysis with Reticulate Trees) v1.7 (Leigh and Bryant 2015) with the epsilon parameter set to zero.

### Microsatellites analysis

Analyses based on microsatellite molecular markers included 43 individuals of both insular and continental origins (Table 1). A panel of 16 microsatellite markers was used and amplified as described previously in Valette et al. (2013) and Vuillaume et al. (2015). Individuals were grouped into five populations according to their sampling localities (i.e., four islands: Montserrat, Saba, Saint Lucia and Grenadines as well as the continental population from French Guiana).

### Genetic diversity

We tested the departures from Hardy-Weinberg expectations and linkage disequilibria using exact tests (1200 permutations) as implemented in the fstat software ver. 2.9.3.2 (Goudet 2001). Significance levels were adjusted for multiple tests using Bonferroni’s standard correction (Rice 1989). We calculated the genetic polymorphism at all loci for each population by computing the allelic richness (Ar), the expected heterozygosity (He) and Fis (Weir and Cockerham 1984) using fstat ver. 2.9.3.2 (Goudet 2001) with 1,200 permutations.

### Genetic structure

**Figure 2.**
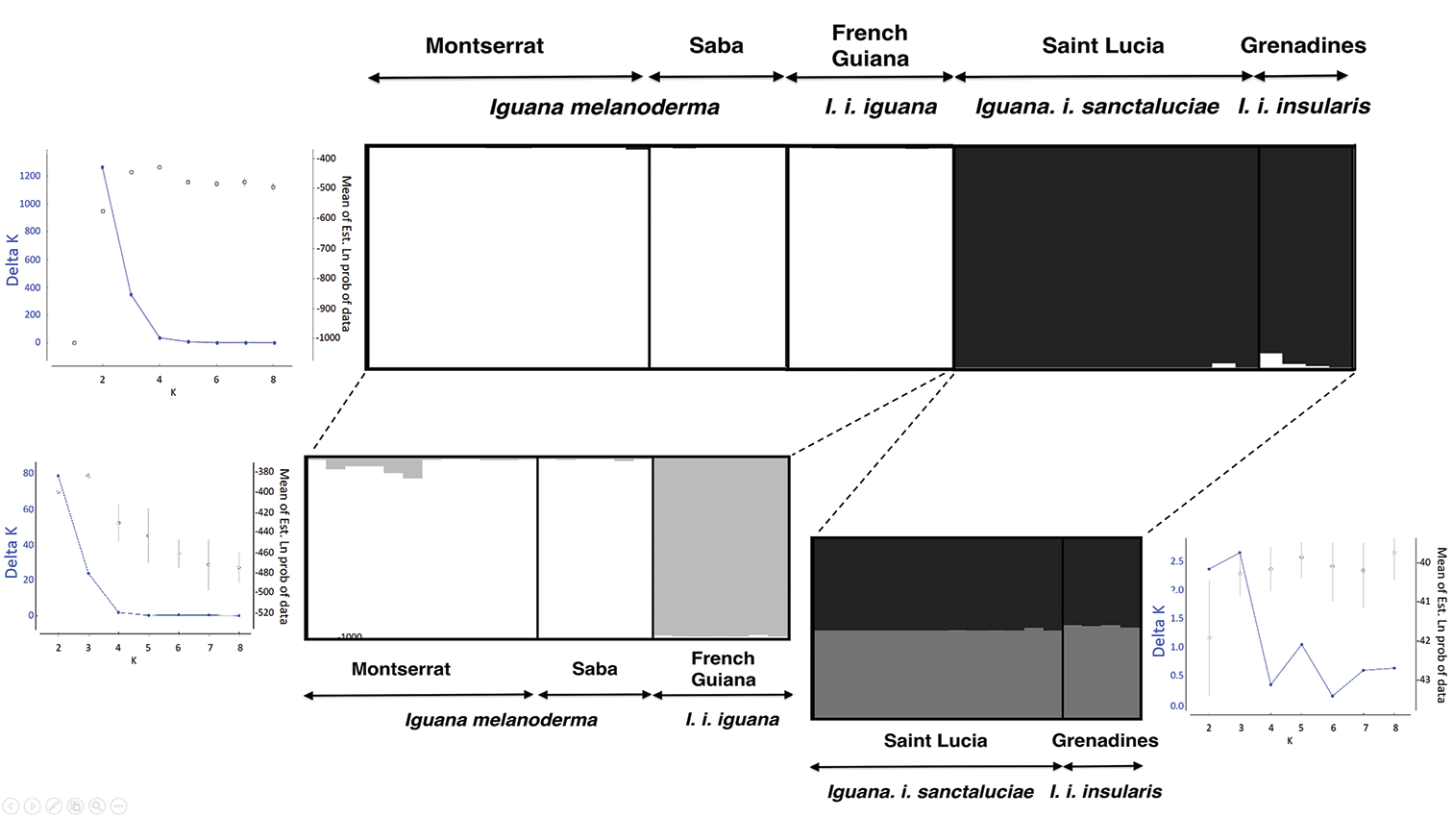
Hierarchical genetic structure of *Iguana* inferred by Structure and Structure harvester. Bar plots show admixture coefficient of each analyzed individuals (represented by each vertical bar) for the inferred genetic clusters K (represented by a different color). The graphs show Delta K values (Evanno et al. 2005) as a function of K (number of clusters) and calculated from posterior probabilities of the data (i.e., ln [P(D|K)]). The results inferred K = 2 genetic clusters when they were initially based on the overall sampling. A subsequent run, including individuals from Montserrat-Saba and French Guiana, revealed significant genetic substructure (i.e., K = 2) separating individuals from Montserrat-Saba and those from French Guiana. Independently, another subsequent run including only individuals from Saint Lucia and the Grenadines revealed no genetic substructure. The bar plots were produced using Distruct 1.1 program (Rosenberg 2004) from the average of the 15 replicates. The names of the taxa are given according to Breuil et al. (2019) and to the conclusion of this work for Saba and Montserrat.

We calculated paired Fst values between populations (Weir and Cockerham 1984) using fstat ver. 2.9.3.2 (Goudet 2001). Their associated significance was computed and tested using global tests implemented in fstat ver. 2.9.3.2 (Goudet 2001). Significance levels were adjusted for multiple tests using Bonferroni’s standard correction (Rice 1989). In addition, we assessed relationships between populations with a Factorial Correspondence Analyses (FCA) based on individual genotypes and using the FCA procedure in genetix v. 4.05.2 (Belkhir et al. 2004). Finally, the population structure was also investigated using the individual-based approach implemented in the structure software (Pritchard et al. 2000). Based on the Bayesian cluster approach, this method allowed the inference of both the number, K, of genetic clusters and the admixture coefficient of individuals to be assigned to the inferred clusters. Initially, we replicated 15 independent runs for each value of K (with K varying from 1 to 9) with a total of 1,000,000 iterations and a burn-in of 100,000. To determine the number of genetic clusters from Structure analyses, we used the Structure Harvester program (Earl and Vonholdt 2012) to compare the mean likelihood and variance per K values calculated from the 15 independent runs (Evanno et al. 2005). The results gave a Delta K value per tested K value allowing the determination of the most likely number of inferred clusters. Subsequent runs were performed to test for the presence of genetic sub-structure within each cluster when K > 1 was initially inferred. These subsequent runs used the same conditions as above (Fig. 2).

## Results

### Population structure and diversity

Based on microsatellite variation, genetic diversity (He) ranged from 0 to 0.82 (Table 2) and allelic richness (Ar) ranged from 1 to 3.86. No linkage disequilibrium was detected (adjusted p-value threshold = 0.0004) and no loci deviated from the Hardy-Weinberg expectations (adjusted p-value threshold = 0.0006). Analyses of microsatellite loci for population structure resulted in the identification of three groups (Fig. 2). Pairwise Fst values ranged from 0.104 to 0.909 (overall Fst = 0.595) and many were significant after Bonferroni correction (Indicative adjusted nominal level (5%) for multiple comparisons was 0.005) (Table 3). These results were corroborated by a FCA procedure which identified the same three groups: one including individuals from St. Lucia and Grenadines, a second including individuals from French Guiana, and the last including individuals from both Montserrat and Saba. Bayesian clustering using Structure and the Structure Harvester programs, also supported population subdivision, giving the highest posterior probability for K = 2. Specifically, individuals from Montserrat-Saba-French Guiana were mainly allocated to the first genetic cluster (i.e., mean ± sd of individual admixture coefficients: 0.997 ± 0.002; 0.998 ± 0.001 and 0.996 ± 0.0001, respectively), whereas individuals from Saint Lucia-Grenadines were allocated mainly to the second genetic cluster (i.e., mean ± sd of individual admixture coefficients: 0.997 ± 0.005 and 0.976 ± 0.029, respectively). Subsequent analyses within each of these genetic clusters revealed further sub-structure separating Montserrat-Saba and French Guiana individuals. In contrast, no genetic sub-structure was detected when considering individuals from Saint Lucia and the Grenadines (whatever the individuals, they showed similar inferences for genetic clusters) (Fig. 2).

**Table 2.** Genetic diversity parameters for each locus. Key: Ar, allelic richness, He, expected heterozygosity, and Fis were computed for each population and loci using FSTAT ver. 2.9.3.2 software (Goudet 2001). No Fis values deviated from Hardy-Weinberg’s expectations (P < 0.0006 after Bonferroni adjustment). NA: not available.

Loci		Montserrat	Saba	Saint Lucia	Grenadines	Guiana	All
**L2**	**Ar**	1.72	1.00	1.00	1.00	1.84	2.98
	**He**	0.23	0.00	0.00	0.00	0.26	0.10
	**Fis**	-0.10	NA	NA	NA	-0.09	-0.10
**L3**	**Ar**	1.00	1.00	1.00	1.00	1.00	1.00
	**He**	0.00	0.00	0.00	0.00	0.00	0.00
	**Fis**	NA	NA	NA	NA	NA	NA
**L5**	**Ar**	1.94	1.91	1.00	1.00	2.00	1.91
	**He**	0.39	0.33	0.00	0.00	0.52	0.25
	**Fis**	-0.29	1.00	NA	NA	-0.09	0.21
**L6**	**Ar**	2.66	1.80	1.00	1.00	2.00	3.36
	**He**	0.53	0.20	0.00	0.00	0.52	0.25
	**Fis**	0.37	0.00	NA	NA	0.73	0.37
**L8**	**Ar**	1.00	1.00	1.00	1.00	1.84	1.18
	**He**	0.00	0.00	0.00	0.00	0.26	0.05
	**Fis**	NA	NA	NA	NA	-0.09	-0.09
**L9**	**Ar**	3.42	2.96	1.00	3.00	2.89	3.80
	**He**	0.66	0.72	0.00	0.75	0.61	0.55
	**Fis**	0.18	0.54	NA	0.33	-0.41	0.16
**L13**	**Ar**	1.00	1.00	1.00	2.00	1.00	1.99
	**He**	0.00	0.00	0.00	0.50	0.00	0.10
	**Fis**	NA	NA	NA	1.00	NA	1.00
**L14**	**Ar**	1.98	2.91	1.36	1.00	1.57	2.65
	**He**	0.45	0.68	0.09	0.00	0.14	0.27
	**Fis**	-0.43	0.02	0.00	NA	0.00	-0.10
**L15**	**Ar**	1.33	1.00	1.00	1.00	2.93	2.33
	**He**	0.08	0.00	0.00	0.00	0.68	0.15
	**Fis**	0.00	NA	NA	NA	0.16	0.08
**L16**	**Ar**	1.00	1.00	1.00	2.00	1.57	1.19
	**He**	0.00	0.00	0.00	0.25	0.14	0.08
	**Fis**	NA	NA	NA	0.00	0.00	0.00
**L17**	**Ar**	1.00	1.00	1.00	1.00	2.52	2.36
	**He**	0.00	0.00	0.00	0.00	0.49	0.10
	**Fis**	NA	NA	NA	NA	0.42	0.42
**L18**	**Ar**	2.41	1.00	1.00	1.00	2.00	1.81
	**He**	0.40	0.00	0.00	0.00	0.53	0.19
	**Fis**	0.45	NA	NA	NA	-0.25	0.10
**L19**	**Ar**	2.91	2.00	1.00	1.00	2.00	3.13
	**He**	0.67	0.53	0.00	0.00	0.52	0.34
	**Fis**	-0.13	0.06	NA	NA	-0.36	-0.14
**L20**	**Ar**	1.98	1.00	1.00	1.00	3.14	3.44
	**He**	0.46	0.00	0.00	0.00	0.66	0.22
	**Fis**	-0.47	NA	NA	NA	-0.09	-0.28
**L23**	**Ar**	1.00	1.80	1.00	1.00	3.86	3.00
	**He**	0.00	0.20	0.00	0.00	0.82	0.20
	**Fis**	NA	0.00	NA	NA	0.30	0.15
**L24**	**Ar**	1.00	1.00	1.00	1.00	1.00	1.00
	**He**	0.00	0.00	0.00	0.00	0.00	0.00
	**Fis**	NA	NA	NA	NA	NA	NA
**All**	**Ar**	1.71	1.46	1.02	1.25	2.07	2.32
	**He**	0.24	0.17	0.01	0.09	0.39	0.18
	**Fis**	-0.03	0.29	0.00	0.50	0.03	0.16

**Table 3.** Fst values for each pairwise population comparison (below the diagonal) and their significance levels (above the diagonal). The indicative adjusted nominal level (5%) for multiple comparisons is 0.005. Fst values that reveal a significant genetic differentiation are in bold.

	Montserrat	Saba	Saint Lucia	Grenadines	Guiana
**Montserrat**	–	0.01	0.005	0.005	0.005
**Saba**	0.104	–	0.005	0.035	0.005
**Saint Lucia**	**0.806**	**0.909**	–	0.005	0.005
**Grenadines**	**0.670**	0.777	**0.555**	–	0.005
**Guiana**	**0.392**	**0.467**	**0.738**	**0.529**	–

### Phylogeny

**Figure 3.**
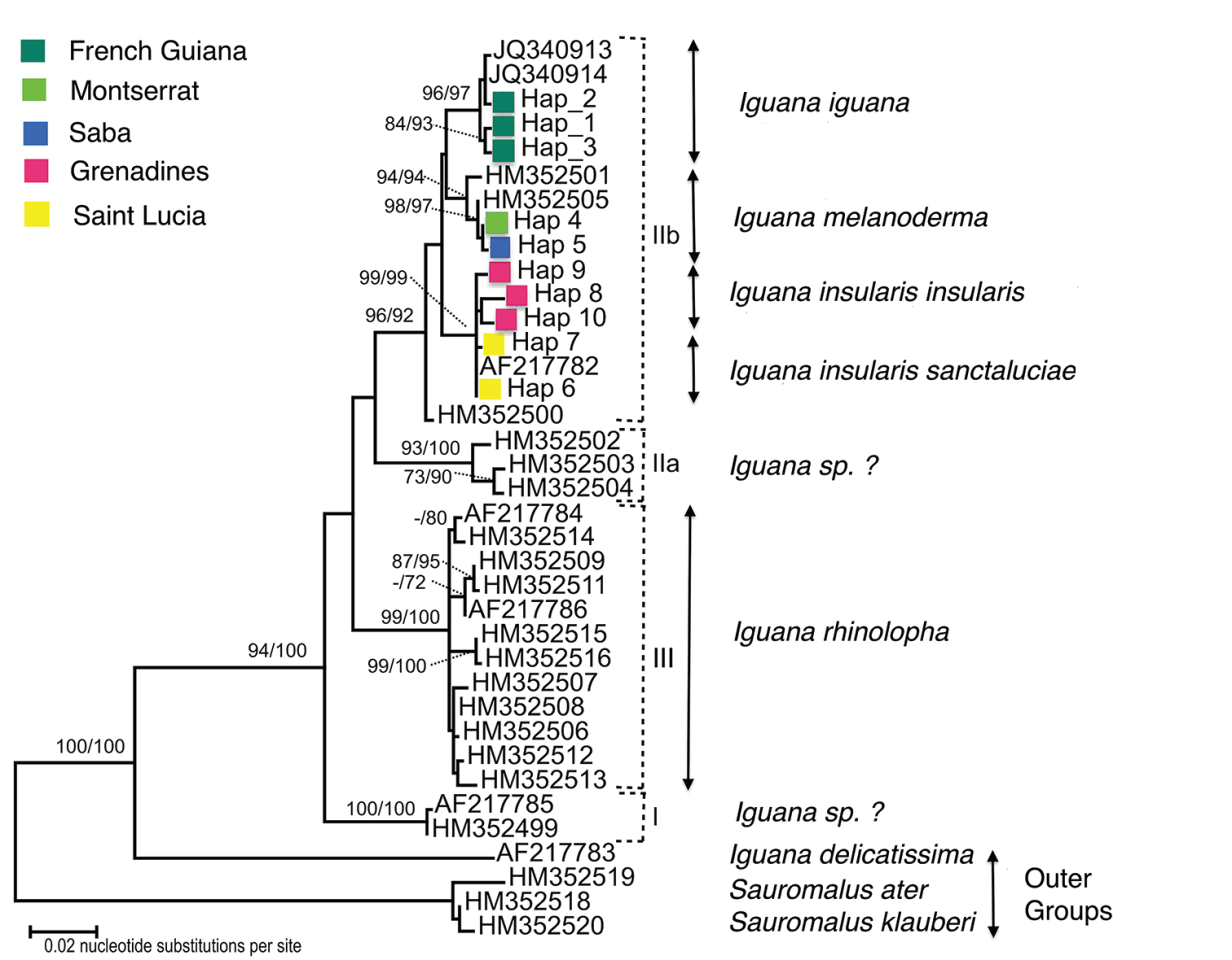
The Maximum Likelihood (ML) tree of the ND4 sequences of iguanas. The percentage of trees in which the associated taxa clustered is shown next to the branches, as bootstrap support (BS ≥ 70) for ML and Maximum Parsimony (MP) topologies respectively. Sequences amplified by the authors of this study were indicated by colored diamond-shaped marks. Marks are colored based on the sampling sites. Sequences from published studies were labelled by their NCBI GenBank access numbers (Fig. 4). The names of the taxa are classified according to the conclusions of this work. Roman numerals refer to clades identified by Stephen et al. (2013).

**Figure 4.**
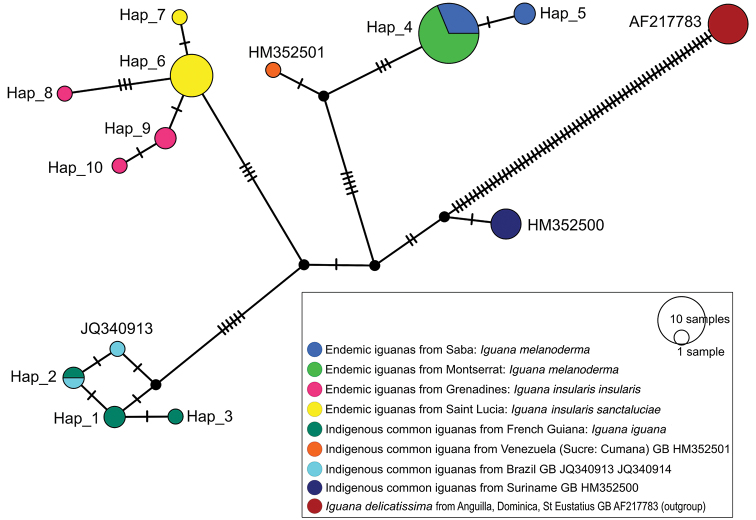
The Median-Joining (MJ) network of the ND4 sequences of iguanas. The sequences amplified by the authors of this study and the corresponding haplotypes from published studies were labelled from “Hap_1” to “Hap_10” (details in Table 4). Other sequences from published studies were labelled by their NCBI GenBank access number. Black circles are median vectors representing extinct or unsampled haplotypes. The remaining colored circles represent haplotypes as nodes in the networks. The circles are colored based on the sampling sites. The size of circles corresponds to the number of specimens with identical sequence. The number of mutational steps is indicated by hatch marks. The names of the taxa are classified according to the conclusions of this work.

The ML tree based on ND4 sequences had the highest log likelihood of – 2861.13 (Fig. 3). The MP analysis, using the same data, generated 2 trees of length 302 (not shown). The consistency index was 0.659, the retention index was 0.892 and the composite index was 0.644 (0.588) for all sites and parsimony-informative sites (in parentheses). ND4 sequences from Montserrat and Saba specimens were placed in a well-supported monophyletic group (Fig. 3; ML BS = 98, MP BS = 97). The Montserrat and Saba sequences were closely related to GenBank sequence HM352501 from an *Iguana iguana* specimen from Venezuela (Sucre: Cumana). In the MJ network (Fig. 4, Table 4), four mutational steps separate HM352501 and “Hap_4” (= HM352505 and MN590163 to 68) encompassed most of samples from Montserrat and Saba (nine in the present study and 13 from published studies). Only two ND4 haplotypes were found to characterise Montserrat and Saba iguanas represented by 24 specimens (9 from this study, 13 from GenBank consisting of 13 from Saba and 11 from Montserrat).

The Saba-Montserrat-Venezuela populations formed a monophyletic group that is the sister group of the populations from French Guiana and Brazil, this group is, in turn, the sister group of the group of St. Lucia-Grenadines that was previously described as two endemic subspecies *Iguana iguana sanctaluciae* for the endemic population of St. Lucia and *Iguana iguana insularis* for the population of the Grenadines (Breuil et al. 2019).

From these genetic data and the following morphological data, it is clear that the endemic iguanas from Saba and Montserrat form a distinct evolutionary entity that we recognize as a new species which is formally describe below accompanied by a taxonomic analysis.

**Table 4.** Haplotypes used in MJ network. Sequences amplified by the authors of this study and corresponding haplotypes from published studies were labelled from “Hap_1” to “Hap_10”. HM352505 haplotype found in the 7 specimens from Montserrat and the 6 specimens from Saba corresponds to “Hap_4”.

Origins of the specimens used for the network	
BRA: Brazil	
GRE: Grenadines	
GUI: French Guiana	
MON: Montserrat	
STL: St. Lucia	
SUR: Suriname	
VEN: Venezuela	
DEL: *Iguana delicatissima* (Anguilla, Dominica, St. Eustatius)	
Hap_1:	2 [IGU78GUI, IGU79GUI]
Hap_2:	2 [IGU82GUI, JQ340914BRA]
Hap_3:	1 [IGU84GUI]
Hap_4:	22 [IGU88MON, IGU89MON, IGU92MON, IGU93MON, SABA01, SABA02, SABA03, SABA04, SABA07, HM352505_M1, HM352505_M2, HM352505_M3, HM352505_M4, HM352505_M5, HM352505_M6, HM352505_M7, HM352505_S1, HM352505_S2, HM352505_S3, HM352505_S4, HM352505_S5, HM352505_S6]
Hap_5:	2 [SABA05, SABA06]
Hap_6:	8 [IGU58STL, IGU63STL, AF217782_1, AF217782_2, AF217782_3, AF217782_4, AF217782_5, AF217782_6]
Hap_7:	1 [IGU65STL]
Hap_8:	1 [IGU73GRE]
Hap_9:	2 [IGU75GRE, IGU76GRE]
Hap_10:	1 [IGU77GRE]
1[JQ340913BRA]; 4[HM352500_1SUR, HM352500_2SUR, HM352500_3SUR, HM352500_4SUR]; 1[HM352501VEN]; 7[AF217783_1DEL, AF217783_2DEL, AF217783_3DEL, AF217783_4DEL, AF217783_5DEL, AF217783_6DEL, AF217783_7DEL]	

### Taxonomic analysis and description

#### Iguana melanoderma sp. nov.

Taxon classificationAnimaliaSquamataIguanidae

3704E4B9-D4A0-54DC-AFD0-5DAC4673F49C

http://zoobank.org/A4984EB3-7F03-412B-8A58-E0CAB36EC836

Figures 5 , 6 , 7

##### Diagnosis.

A species of *Iguana*, with a distinctive melanistic phenotype, with a black dewlap, huge tubercular nape scales, the absence of horns on the snout.

##### Etymology.

The name was chosen to emphasize the most conspicuous feature of this new taxon, from *melano* meaning black and *derma* meaning skin.

Common local names are: Melanistic Lesser Antilles iguana, Saban Black iguana

##### Type material.

***Choice of the holotype and the paratype.*** The choice of our type specimens and the way we conducted the description of the type and the paratype attempt to best meet the criteria proposed by (Dubois 2009) for the case of *Conolophus marthae* described by Gentile and Snell (2009). We took into account the same kind of problems of sacrificing a specimen for museum collection and referencing an emblematic, iconic, large lizard that belongs to an endangered taxon.

(1) The Saba and Montserrat populations survived for a long time in a period when the risk of extinction was lower than today. Nevertheless, the iguanas were hunted for food, killed by cats and dogs, and their habitats destroyed by livestock and natural events. Today, in Saba, the main risk for this small endemic population, which seems to be far from the carrying capacity of the island, is the arrival of invasive common iguanas from South and Central America that have a rapidly expanding population in Saint Maarten. In Montserrat, the same risk exists. Volcanic eruptions are also a major threat to these populations, as evidenced by the eruption of the Soufrière Hills in Montserrat in 1995 and the following years, which destroyed about a third of the island.

Hybridization with closely related lineages may in-fact be the greatest risk and could very likely lead to extinction of endemic lineages, as is the case for *Iguana delicatissima* in Guadeloupian Archipelago (Vuillaume et al. 2015). Moreover, the description of a new taxon may attract collectors and lead to unintended and undesirable consequences. This is of concern because on the Dutch island of Saint Maarten, both the legal and illegal pet trades are common. With the description of this new taxon, local authorities, such as the *Saba Conservation Foundation* and the NGO *Sea and Learn*, will have tools to protect the Saban Black Iguana from poaching on an island where the terrestrial protected area is less than 0.5 sq. km on the edge of this endemic iguana’s range.

(2) Lazell (1973) studied and collected iguanas in Saba and Montserrat in the 1960s. The vouchers are deposited at the Museum of Comparative Zoology (Harvard) with two other individuals collected in the seventies. Six vouchers for these two islands (Saba: MCZ R-75832, R-75833, R 133096; Montserrat: MCZ: R-61119, R-82310, R-126377) are present but unfortunately it is almost impossible to measure them properly due to their poor condition. In addition, some are young individuals, which do not have well developed diagnostic characteristics.

(3) We have no precise idea of the size of the iguana population in Saba and Montserrat. Rough estimates based on density in some surveyed areas yield 100–300 adults and subadults for each island. In theory, it is always possible to catch a senile non-breeding male and prepare it in good conditions act as a voucher that will be available in a Museum collection for future study. But, for technical reasons, when the first author was in Saba in 2012, it was not possible to collect such an individual. Roadkill animals are often in poor conditions (broken, flattened, rotten) and in most cases cannot be studied and preserved in a zoological reference collection. The same remarks apply to the Montserrat population.

(4) Since diagnostic characteristics are mainly visible in adult individuals and are not measurements, we chose for the holotype of this new taxon specimen MCZ R-75032 from Saba collected by JD Lazell 6/23/63 on the Windward side of Saba. The paratype MCZ R-126377 is a head of an adult from Montserrat collected by JO Boos at Old Road Bluff 8/6/1970. These two samples are housed at the Museum of Comparative Zoology (MCZ, Harvard). They present the diagnostic characters as described by (Breuil 2013, 2016) and represent the populations of the two islands, respectively.

##### Holotype

**Figure 5.**
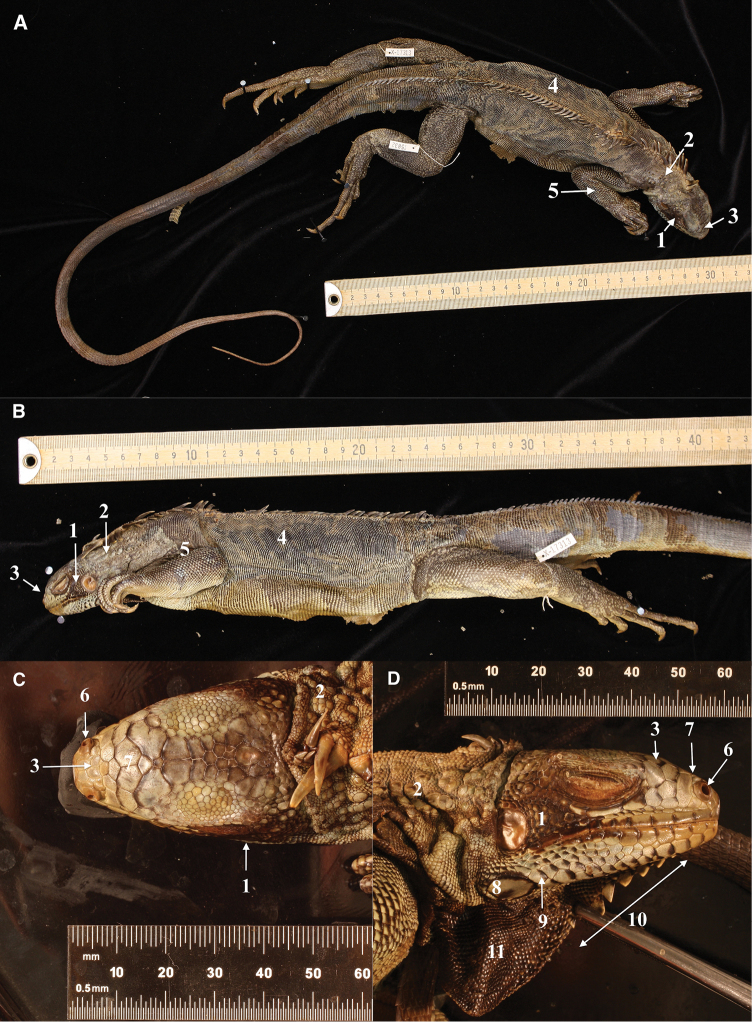
Holotype of *Iguana melanoderma* sp. nov. **A** Dorsal view **B** lateral view **C** dorsal view of the head **D** lateral view of the head. MCZ R-75832 from Saba, Windwardside. Museum of Comparative Zoology, Harvard University. President and Fellows of Harvard College. 1. Black patch between the tympanum and the subtympanic plate. 2. High number of aligned nape tubercles. 3. No horns on the stout. 4. Dorsal carpet pattern. 5. Black on the upper part of the forelimb. 6. Prominent nostrils. 7. Anterior part of the snout, not black. 8. Subtympanic plate with a dark posterior patch. 9. Black anterior edge of lower sublabial scales. 10. Fewer than 10 triangular gular spikes extended in the upper part of the lower dewlap. 11. Entirely black dewlap.

(Fig. 5). Lesser Antilles, Saba• ♀; Windward side; 23 June 1963; JD Lazell [leg.]; MCZ (R-75832).

This individual from Saba was not measured in detail because of the risk of spoiling the specimen. It is an adult female of approximately 26.3 cm SVL and a tail length of approximately 66.4 cm and a total length of 92.7 cm (measurements and photographs by Joseph Martinez, MCZ, Harvard).

##### Description of the holotype.

The subtympanic plate is round, with a dark patch in the posterior part. The anterior, upper, and lower parts of the subtympanic plate are surrounded by black pigment. Most labial and sublabial scales have dark coloration on their anterior side. The lower labial scales, before the subtympanic plate, are arranged in a series of five pairs of scales of quite similar size and located one in front of the other. The tubercular nape scales are numerous, well developed, grey, and aligned in rows.

##### Color pattern.

This specimen is partially discolored, with a slight carpet pattern, the stripes on the tail are almost invisible in the photographs, but according to (Lazell 1973) who captured this specimen, it had a conspicuous carpet pattern.

The dewlap is black in its lower part, and there are nine small, triangular yellowish gular spikes. The tympanum is brown. There is a conspicuous black spot between the eye and the tympanum. The snout and the top of the head are light, not black. The dorsal spikes are greenish and black.

##### Paratype

**Figure 6.**
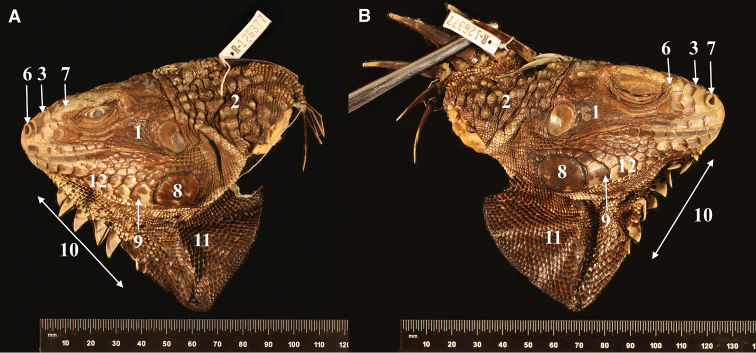
Paratype of *Iguana melanoderma* sp. nov. **A** Left side view of the head **B** right side view of the head. MCZ R 126377 from Montserrat, Old Road Bluff. Museum of Comparative Zoology, Harvard University. President and Fellows of Harvard College. 1. Black patch between the tympanum and the subtympanic plate. 2. High number of aligned nape tubercles. 3. No horns on the stout. 5. Black spot on the upper forelimb. 6. Prominent nostrils. 7. Anterior part of the snout, not black. 8. Subtympanic plate with a dark posterior patch. 9. Black anterior edge of lower sublabial scales. 10. Fewer than 10 triangular gular spikes extended in the upper part of the lower part of the dewlap. 11. Dewlap entirely black. 12. Lower sublabial scales arranged in pairs of nearly the same size.

(Fig. 6). Lesser Antilles, Montserrat • ♂; Old Road Bluff; 6 Aug. 1970; JO Boos [leg.]; MCZ R-126377.

##### Description of the paratype.

This individual is only a head of a small adult male based on the size of the dorsal spikes. This head presents the typical characteristics of this taxon: large grey scales on the tubercular nape, black spot between the eye and the tympanum, and labial and sublabial scales with black patches on the anterior part. There are five pairs of scales before the subtympanic plate almost completely black, a black dewlap, and a flat head with a light snout (photographs by Joseph Martinez, MCZ Harvard).

##### Type locality.

On the Windward side of Saba for the holotype and on Old Road Bluff, west coast of Montserrat for the paratype (Figs 1, 11).

##### Description of *Iguana melanoderma*.

*Iguana melanoderma* is distinguished from all other iguana lineages by the following combination of characteristics. This description is mainly based on adult iguanas observed in the field with the most developed diagnostic characteristics.

**Figure 7.**
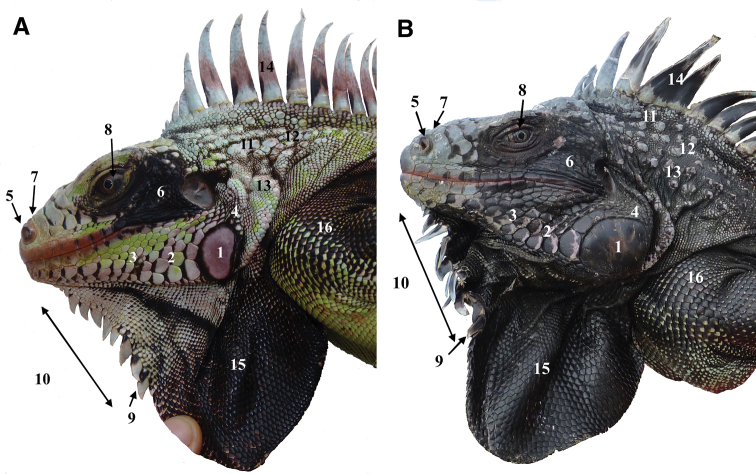
Comparison of morphological features of the head. **A** Young adult male. 1. Subtympanic plate with pink in the center. 2. Lower sublabial scales arranged in pairs of nearly identical size. 3. Black anterior edge of the lower sublabial scales. 4. Black border around the subtympanic plate. 5. Prominent nostrils. 6. Black spot between the eye and the tympanum. 7. Absence of horn and light snout. 8. Dark brown eye. 9. Triangular gular spikes. 10. Fewer than 10 gular spikes extended in the upper part of the lower dewlap. 11. High number of aligned nape tubercles. 12. Prominent light-grey tubercles. 13. Light greyish-green coloration on the neck. 14. High light-grey dorsal spines. 15. Dewlap half black. 16. Dorsal part of the limb with light-green scales becoming black with the extension of melanin from the anterior edge to the posterior edge of the scales. **B** Old male of *Iguana melanoderma* (Saba). 1. Large all-black subtympanic plate. 2. Extension of the black pigment on the sublabial scales. 3. Black coloration of the labial and upper sublabial scales. 4. Black coloration between the tympanum and the subtympanic plate. 6. Extension of the black spot around the eye and on the posterior labial and sublabial scales.7. Snout turning dark grey. 9, 10. Gular spikes turning dark grey with extension of black patches. 12. Dark grey nape tubercles. 13. Black coloration on the neck. 14. Dorsal spikes turning black. 15. Dewlap completely black. 16. Black upper face of the limb.

*Iguana melanoderma* belongs to the Common Green Iguana phenotype (in contrast to the *Iguana delicatissima* phenotype) with its large subtympanic plate, the arrangement of sublabial scales, the rectangular shape of the dewlap, the shape and the distribution of the gular spikes, its flat head, its tubercular nape scales, and its banded tail (Breuil 2013, 2016). By the absence of horns on the snout, *Iguana melanoderma* can be distinguished from *Iguana rhinolopha* and *I. iguana insularis*, and from *I. iguana sanctaluciae* (Fig. 7).

**Figure 8.**
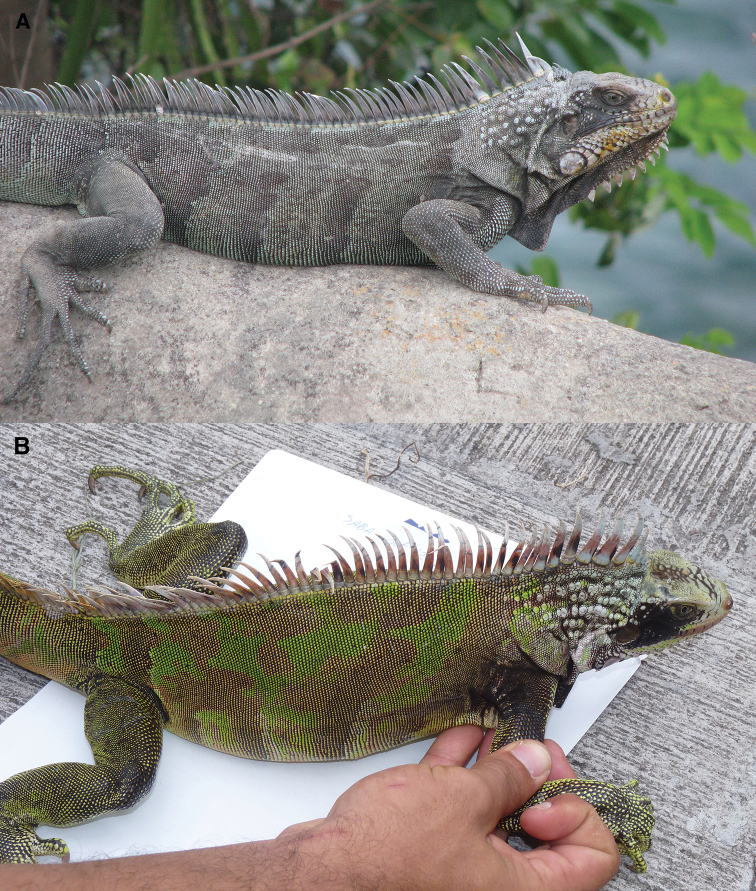
The dorsal carpet pattern of young adults *Iguana melanoderma*. **A** Montserrat **B** Saba. The dorsal coloration is formed by darker more or less interrupted dorso-ventral bands (brown, dark grey) on a lighter ground. This pattern disappears in old individuals. The black patch between the eye and the tympanum is already visible.

The most distinctive morphological trait of this new taxon is its general color: adults from Saba and Montserrat iguanas are melanistic. There is a tendency for individuals to become blacker with age (Fig. 8).

There is always a black spot between the brown to grey-brown eye and the tympanum. In fully grown adults, the subtympanic plate is 2 to 2.5 times the height of the tympanum. Its color varies from light pink to dark pink with a proportion of black coloration ranging from hardly black to all black. The tympanum can be completely black. The labial and sublabial scales have the same coloring as the subtympanic plate. The lower labial scales, anterior to the subtympanic plate, are arranged in 3–5 pairs of scales of fairly similar size, one in front of the other, and do not form a mosaic of small scales. The head is usually black on the sides (tympanum, eye, subtympanic and posterior labial and sublabial parts), whereas the snout and the top of the head are light to dark grey, and in some individuals these parts are nearly entirely black.

The dewlap is completely black in adults, as in *Iguana iguana sanctaluciae* (Fig. 7). The gular spikes are light to dark grey with a variable portion of black. They are flat, triangular, quite small, and not exceed 10 in number. A variable percentage of the gular scales on the lower part of the dewlap are pentagonal or hexagonal, and do not overlap.

The dorsal parts of the limbs are more or less black, and the black is more developed in older individuals extending over the ventral face of the limb. Some specimens have entirely black head and legs whereas the body is dark green. This body coloration is the result of a black anterior part and a lighter posterior part of most scales while some others are black or dark green. The spikes of the dorsal crest range from light to dark grey; the central part can be black. Some individuals have entirely black dorsal and caudal crests.

The nuptial coloration is present in both sexes, but more vivid and more developed in males than in females. Breeding adults sometimes become reddish-orange over the entire body (Powell et al. 2005) and the jowls are pinkish if not too melanistic. They never become as orange as *I. rhinolopha*. According to Lazell (1973), in melanistic individuals, the face, snout, and sometimes the sides are usually purple or brown.

**Figure 9.**
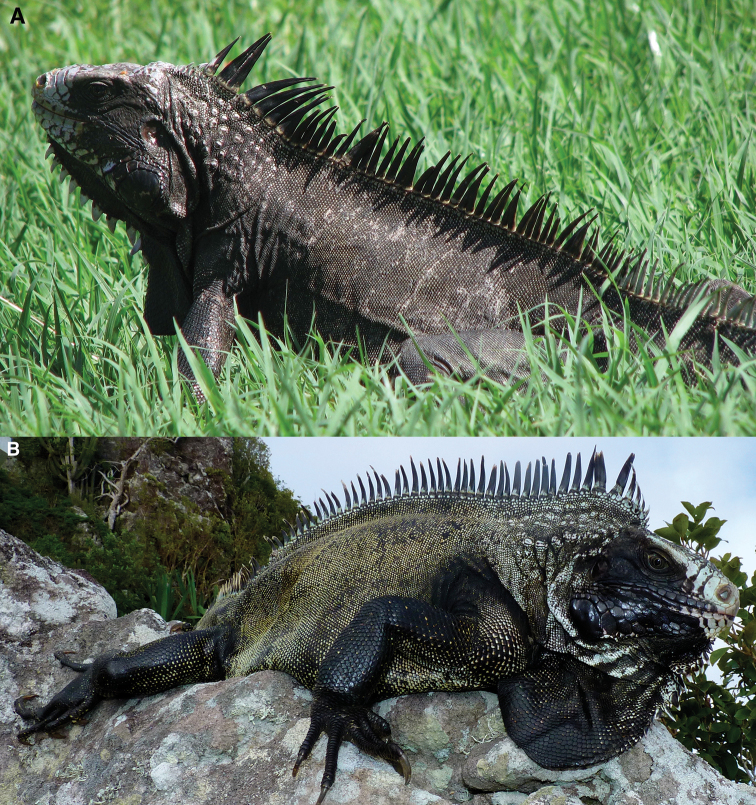
**A** Old adult from Montserrat **B** old adult from Saba. In these old individuals the carpet pattern is absent. The head is almost entirely black, except for the top and the snout. The dewlap, neck, dorsal spikes, and forelimb are black. Dorsal and lateral coloring is more variable, ranging from entirely black to a mosaic of black, brown, and dark green scales.

The iguanas from Saba and Montserrat begin their lives with discontinuous light, medium and dark green dorsolateral bands and patches, some of which are underlined by white markings without black on the head and limbs. The black spot between the eye and the tympanum is present in one-year-old individuals, but it is very small and poorly developed. The proportion of the areas covered by these different green markings varies in hatchlings. In juveniles and subadults, this pattern then gradually changes to an ornate arrangement, called a carpet pattern by Lazell (1973) which consists of interrupted bands and patches, green and brown or grey and green, according to the skin shade (Fig. 9). This highly disruptive carpet pattern may be the mark of an ancient adaptation to crypsis. A light carpet pattern, with brown and green, is also sometimes present in adults. With age, the individuals become darker, causing the carpet pattern to fade. The granular scales on the body are green, but at their periphery they are black, and a varying proportion of these scales are completely black. The details and chronology of these ontogenetic transformations are unknown.

There are no nasal horns. The tubercular nape scales are numerous, prominent, ranging from light to dark grey, and are often aligned in many rows. The cheek scales usually do not overlap.

Montserrat iguanas are similar to those of Saba. Overall, they appear less melanistic, but some individuals are as black as those of Saba. The head appears to be flatter and more elongated in Montserrat than in Saba, but more data are needed to assess putative morphological divergence between the two populations.

##### Biology.

**Figure 10.**
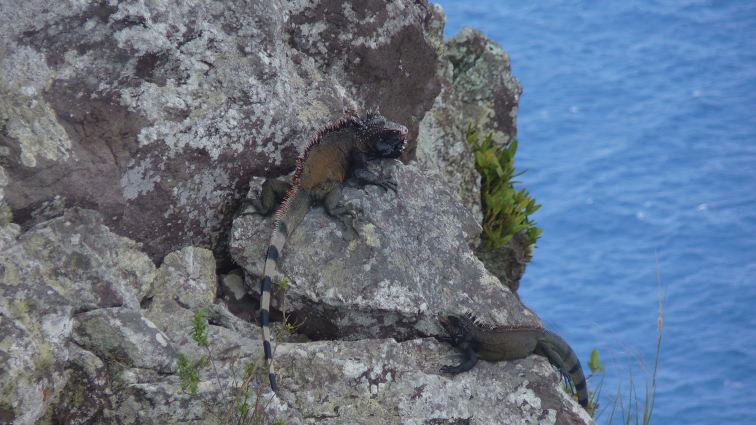
*Iguana melanoderma* sunbathing at dawn on the Windward coast of Saba.

**Figure 11.**
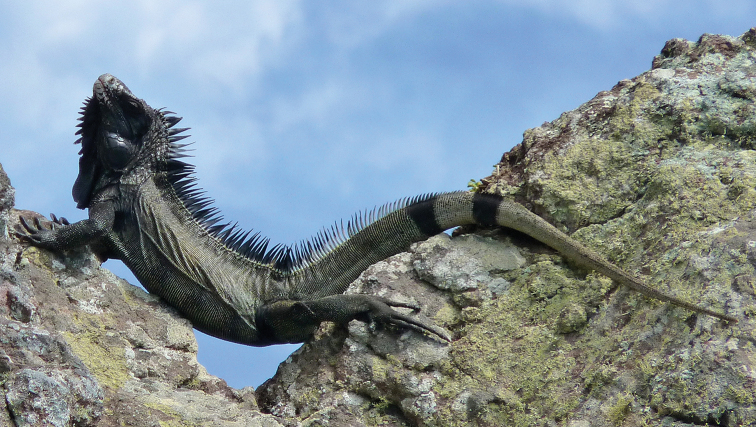
A basking *Iguana melanoderma* optimizing after different trials its warming by a curved position when the sun is low on the horizon on the Windward coast of Saba.

In Saba, *Iguana melanoderma* lives on cliffs (Fig. 10), in trees and bushes, in shrublands, and deciduous woodlands. One of the most striking facts about Saba is that these iguanas live in a foggy and cool environment up to about 500 m a.s.l. They sunbathe as soon as the sun rises (Figs 10, 11). The black coloration may be an adaptative trait to help rapidly raise their body temperature in these cool conditions (Breuil 2013, 2016). Hatchlings were observed in June and July.

In Montserrat, *Iguana melanoderma* have been reported in a variety of habitats, mostly in coastal residential areas. In 1995, before the eruption of the Soufrière Hills, iguanas could be seen in the then capital, Plymouth, along the seawall defenses just above high tide (M. Morton, personal observation). Blankenship (1990) reported a preference for ghauts (streams), and the majority of records to date are from locations near ghauts or rivers (J. Dawson, SL Adams, pers. comm., E. Corry, pers. obs.).

In Montserrat, there are far fewer records from mesic forest (Hamilton et al. 2008) from the Centre. The highest elevation for iguana sightings has been cited as ca. 400 m a.s.l. (G. Garcia pers. comm). However, this may reflect a bias towards areas where people spend the most time. Fewer people visit the forests, and far fewer people travel to the east of the island after the eruption. That being said, Daltry (1995) reported a negative association between iguana sightings and the elevation of the study plot (P < 0.0023).

According to Blankenship (1990), Montserrat iguanas breed from late February, when nest digging begins, until the emergence of hatchlings in July and August. Clutch sizes range from 15 to 30 (Blankenship 1990). Observations after 1995, i.e., after the start of the last eruptions cycle of the Soufrière Hills, indicate that nesting has continued at these times of the year; egg-laying was recorded in March and April (E. Corry, M. Morton, personal observations). In April 2008, one female was observed nesting in sand at Iles Bay, an area of recent lahar deposits at the mouth of the Belham River, as well as higher up in this now subterranean stream (E. Corry; M. Morton, personal observations). This is consistent with Blankenship (1990), who stated that they nest in loose soil.

##### Distribution.

**Figure 12.**
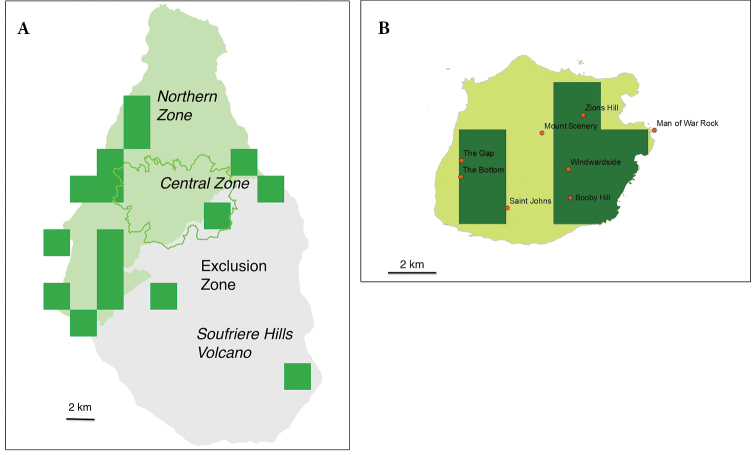
Distribution of *Iguana melanoderma* in Montserrat and Saba. Each square is 2 km along a side.

The volcanic island of Montserrat is 102 km^2^. In 1995, the dormant volcano of the Soufrière Hills became active. Catastrophic eruptions in 1997 rendered two thirds of the south of the island uninhabitable and led to the creation of an exclusion zone (Fig. 12A). There are three major mountain ranges with natural vegetation restricted to small areas on the tops of two. The highest point before the eruption was Chances Peak, which reached 914 m a.s.l. The subsequent lava dome naturally rises and falls periodically; its maximum height was 1050 m a.s.l. in 2015. According to Lazell (1973), in the 1960s, iguanas were locally abundant in southern Montserrat and were present throughout the lowlands of the island. Steadman et al. (1984) reported that the species occurs in scattered areas around the island, and is locally common in some places along the southern coast. According to Reitz (1994), the iguana was not common on the island.

The island of Saba is about 13 km^2^ and rises to an altitude of 877 m a.s.l. on Mt Scenery. This peak forms the summit of a dormant 400 ka-year-old volcano (Roobol and Smith 2004). Saba is surrounded by steep cliffs on all sides. There is no permanent beach for the laying of iguana eggs as in Martinique around Mt Pelée. Much of the central highlands of the island, above 400–500 m, are covered with dense primary and secondary rainforests. Rain and moisture from the surrounding clouds bring humidity to the forest. This pristine habitat thus seems to be incompatible for a permanent presence with the thermal and solar needs of iguanas, even in the canopy where clouds and mist are often present.

The superposition of the geological map (Roobol and Smith 2004) and the current vegetation shows that the rainforest is developing on the andesite lava of the recent central volcano. On the pelean volcanic domes on the periphery of Mount Scenery, patches of xeric vegetation are found, as on Lower and Upper Hell’s Gate, Level, and Great Hill. Some hills, such as Old Booby, have little tree vegetation; overgrazing by goats is responsible for this. Some of the cliffs are made of volcanic tuff which is a poorly consolidated material that cannot withstand heavy tropical rains and where trees cannot grow. The vegetation and the climatic conditions, temperature and sun, therefore seem to restrict the distribution of iguanas and thus their numbers.

As such, the Saban Black Iguana is mainly present on the Windward side, from sea level to about 500 m a.s.l. (hilltop at the Level 514 m) (Fig. 12B). The main concentration is found on the slopes of Lower and Upper Hell’s Gate, and on the cliffs of Booby Hill. The total range of this taxon is about 5–6 km^2^. There are also some iguanas at The Bottom. This locality is located west of Windwardside village, but not in the shadow of Mt Scenery. For Lazell (1973), in the 1960s, Saba iguanas were common everywhere, even at 800 m a.s.l. in the rainforest of Mt Scenery.

## Discussion

### Morphological remarks

In the US Virgin Islands (St. Thomas, St. Croix, St. John) and the British Virgin Islands (BVI: Tortola) as well as on the coastal islands of Venezuela, and on the coast in the vicinity of Cumana, there are also melanistic iguanas (Lazell 1973; MacLean 1982; Buurt 2005; Falcón et al. 2018), but we found no morphological and color differences from the melanistic iguanas of Saba-Montserrat. Thus, as a hypothesis, we consider here that these melanistic iguanas belong to the same lineage as *I. melanoderma* (see below).

### Distribution remarks

Such a discontinuous distribution of this new species is puzzling. It can be explained by: [1] the natural dispersal from northern Venezuela (mainland and coastal islands) where iguanas with the same melanistic phenotype are found, as in Margarita, Los Roques and La Blanquilla, [2] human dispersion by pre-Columbian Indians, [3] recent dispersal by Modern Man, [4] convergent evolution, [5] the regression of a wider distribution area, or [6] a combination of the previous hypotheses.

To successfully colonize an island, iguanas must first arrive on that island by natural or human means, occupy an available ecological niche, or if not, be able to successfully compete with the local species. Based on what we know about the competition between *Iguana delicatissima* and *Iguana iguana* (Breuil 2013, 2016; Vuillaume et al. 2015), it is likely that *Iguana melanoderma* first became established in Montserrat-Saba and prevented the subsequent establishment of *Iguana delicatissima*. We found no evidence of mitochondrial introgression with I. *delicatissima* in this new taxon, whereas we discovered mitochondrial introgression with I. *delicatissima* in *Iguana iguana sanctaluciae* (Breuil et al. 2019). Vuillaume et al. (2005) found no evidence of introgression with microsatellites in the Saba population. The possibility of nuclear introgression should be investigated in Montserrat to test the hypothesis of an ancient presence of *I. delicatissima*.

The first iguanas were collected in Saba by F. Laglois in 1879 (Garman 1887) and were housed at the MCZ (Harvard), but they no longer appear to be present according to the database of this museum. The first mention of iguanas in Montserrat seems to be recent, as this species is not mentioned by Dunn (1934) or by Barbour (1937), although herpetologists, such as Garman (1887), visited the island from where he described the endemic species *Anolis lividus*. This does not mean, however, that there were no iguanas on this island at that time. The first mention of iguanas in Montserrat was probably published by Underwood (1962).

Saba and Montserrat are separated by 150 km. Between the two is the Christopher Bank or St. Kitts Bank (St. Eustatius, St. Kitts, Nevis) which was inhabited in historical times by *Iguana delicatissima* perhaps with the exception of St. Kitts (Lazell 1973). Nowadays, only a small population of *Iguana delicatissima* is present in St. Eustatius (Van den Burg et al. 2018b) and St. Kitts and Nevis have no *Iguana delicatissima*. In this context, the presence of *Iguana melanoderma* in Saba and Montserrat appears to be an anomaly in respect to the historical distribution of *Iguana delicatissima*. This species has inhabited in historical times, with the exception of these two islands, all islands from Martinique to Anguilla. This can be explained if *Iguana delicatissima* extended its geographical range with the help of Amerindians after the arrival of *I. melanoderma* in Saba and Montserrat.

Steadman et al. (1984), Reitz (1994), and Pregill et al. (1994) reported the presence in two ceramic assemblages in Montserrat (± 2500 years ago) of Iguana cf. iguana bones that were used with other vertebrates as food. In Montserrat, the iguana is believed to have been introduced by Amerindians (Young and Hilton 2008) while Underwood (1962) thought it was a recent introduction but at that time no archeo-zoological iguana remains were known. Edgar (2009) suggested that the population may be native to Montserrat but that further studies are needed.

In Saba, a pre-ceramic occupation dating from 3300 BP with dense midden deposits, mainly land crabs and birds, located at 400 m a.s.l., in the tropical forest, was studied by Hofman and Hoogland (2003), but did not reveal any iguana remains. For these authors, the use of these limited food resources and the nature of the tools suggest a temporary seasonal occupation. Other Amerindian sites on Saba are dated between 400 and 1400 AD, with a major occupation period between (800–1400 AD) (Hofman and Hoogland 2003). These latter coastal archeological sites contain *Iguana iguana* bones (Hofman and Hoogland 1991), but it appears that this attribution is based only on the fact that *Iguana iguana* is the species present today on this island.

The similarity in morphology and coloration of these two populations (Breuil 2013, 2016) and their genetic homogeneity with respect to the ND4-Leu sequence and microsatellites suggest that an introduction from Saba to Montserrat or vice versa during the Amerindian period is a viable hypothesis. Our results clearly show that this Montserrat iguana belongs to the same group as Saba. The iguana bones in the Saba and Montserrat refuse middens strongly indicate that, while the attribution of bones to *Iguana iguana* by Hofman and Hoogland (1991) is correct with respect to *delicatissima*, Iguana cf. melanoderma is associated with Amerindians artifacts. However, this does not exclude the possibility of natural and/or Amerindian introductions from the eastern coastal region of Venezuela.

According to Schmidt and Inger (1957): “The common iguana, *Iguana iguana*, is apparently a recent immigrant to the southern Lesser Antilles and the Virgin Islands from northern South America, and has driven out the native rock iguana (genus *Cyclura*…) from these islands”. Platenberg and Boulon (2006) suggested that this species was introduced to the latter islands by the pre-Colombian Indians, possibly to replace the native rock iguana as a food source, although it may also have floated or rafted there. According to Mayer (2012), all *Iguana iguana* present in the Virgin Islands were introduced. Recent introductions of iguanas from the pet trade into the USVI may have occurred from different locations (Falcón et al. 2013). According to Falcón et al. (2018), haplotypes of Caribbean iguanas were found on the islands of Vieques (Puerto Rico Bank) and St. Croix (USVI). These authors also found that all individuals from St. Thomas (USVI) with the melanistic phenotype shared haplotypes found in iguanas native to two Caribbean Islands not mentioned in their study. These preliminary genetic data concerning the melanistic iguanas of the Virgin Islands and Puerto Rico Bank and their morphology, which is indistinguishable from the iguanas of Saba-Montserrat, suggest that these melanistic iguanas belong to *I. melanoderma*. Genetic studies with microsatellites for these populations are urgently needed to answer this question in view of the presence of invasive iguanas (*Iguana iguana iguana*, *Iguana rhinolopha*) on the Virgin Islands and Puerto Rico Bank.

The Pleistocene natural deposits of Marie-Galante do not contain the remains of *Iguana delicatissima* nor *Iguana iguana* while many other reptiles, now extinct, are present. *Iguana delicatissima* appears on this island with Amerindians artifacts that suggest human introduction (Grouard 2001; Bochaton et al. 2015). The current distribution of *I. delicatissima* is thus a mixture of natural colonization and Amerindian introductions as it is now demonstrated.

Based on our data and those of Stephen et al. (2013), we propose that the origin of these insular populations with the black phenotype results from a first colonization from coastal Venezuela (Cumana area) and the Venezuelan continental islands such as Margarita, Los Roques or La Blanquilla where melanistic iguanas are present to Saba or Montserrat. A pre-Columbian colonization from one of these two islands to the other may have followed, and a third colonization towards the Puerto Rico Bank and St. Croix Bank. The original differentiation leading to this black phenotype and genetic differentiation may have taken place in the Cumana region. The colonization of Saba-Montserrat and the Puerto Rico Bank, Virgin Islands, and St. Croix Bank, with little or no divergence, may be the consequence of multiple, diachronic, Amerindians or recent colonization events.

### Phylogeographic remarks

Stephen et al. (2013) suggested that there were two natural dispersal events in the Lesser Antilles. The older one gave birth to the population of St. Lucia which is the most differentiated and is now considered as a subspecies *I. iguana sanctaluciae* (Breuil et al. 2019), and the most recent event to the Saba-Montserrat population, less differentiated from the Venezuela population, considered here as *I. melanoderma*. The phylogeny of Stephen et al. (2013) proposed the existence of three lineages in the Common Green Iguana. The deepest and therefore the oldest is represented by the Curaçao population which, in this context, warrants a specific status. The intermediate lineage is represented by the Central American clade and the most recent is the South American lineage to which the insular populations of St. Lucia-Grenadines and Saba-Montserrat-Venezuela belong. Breuil (2013, 2016) proposed to consider the Central American iguana clade as *Iguana iguana rhinolopha* and the South America clade as *Iguana iguana iguana*. Breuil et al. (2019) upgraded these two subspecies to the species level.

The five differences (5/818 = 0,6 %) between the haplotype (HM352505) of Saba-Montserrat and the haplotype of Venezuela (HM352501) suggest differentiation, with a genetic divergence, approximated by a molecular clock of 1.29 million years for every 1% sequence divergence, at the ND4-Leu Locus (Malone et al. 2000), of ka. However, if the dark coloration of *I. melanoderma* is an adaptation to the cold environment of Saba, we have no parsimonious explanation for the presence of melanistic iguanas in northeastern Venezuela, and on the St. Croix and Puerto Rico Banks, except a very recent diaspora in a context where this black coloration does not show any loss of fitness in a very safe and protective environment.

Hap_4 (GB HM352505), the most common haplotype, is shared by these two insular populations, but Saba also has its own haplotype (Hap_5) (Fig. 4). This most common haplotype is, according to Falcón et al. (2018), also present in the Puerto Rico Bank. This is a strong argument in favor of a recent diaspora of this melanistic iguana throughout the Caribbean Region. A similar situation was found in *Leptodactylus fallax* where no genetic differences were found between the Montserrat population and the Dominican population (Hedges and Heinicke 2007). These authors suggested that Amerindians were responsible for this distribution pattern even though it had been present in the past in St. Kitts, Martinique, and St. Lucia (Lescure 2000).

Montserrat consists of three major volcanic centers: to the north are the heavily eroded Silver Hills (ca. 2.6–1.2 Ma); in the center are the Centre Hills (ca. 950–550 ka), also extinct and crossed by deep erosive canyons; and to the south is the massif comprising the South Soufrière Hills (ca. 135–125 ka) and the Soufrière Hills (ca. 170 ky to present) (Harford et al. 2002). Based on geographical distance and age, Montserrat is a better candidate for first natural colonization by propagules from Venezuela. Montserrat is located 150 km southeast of Saba.

Saba’s oldest rocks are about 400 ky years but most of the volcanic deposits were produced in the last 70,000 years and have increased the size of the island on the edge of Mt Scenery. The last eruption is dated to 280 years BP and covers Amerindian artifacts but underlies those of the European settlers who colonized the island in 1640. European settlers may have been attracted to the island because of the presence of grassland instead of tropical rainforest caused by an eruption shortly before European settlement (Roobol and Smith 2004). Under these conditions, a recent major bottleneck may have occurred in the iguana population.

A bottleneck hypothesis and/or founder effect to explain the low genetic diversity of these insular iguanas is congruent with the fact that of the 16 common microsatellite loci used in this study, 9 are monomorphic in Saba while 8 are monomorphic in Montserrat with twice as many individuals. For example, the *Iguana delicatissima* population of Petite Terre (Guadeloupian Archipelago) has 8/15 monomorphic microsatellite loci and the Chancel Islet population 6/15. In both populations, serious bottlenecks have recently occurred (Breuil 2002; 2009). Indeed, this panel of microsatellite molecular markers revealed high polymorphism in all the other common iguana populations studied by Valette et al. (2013) and Vuillaume et al. (2015).

Results based on microsatellite markers revealed a higher level of genetic diversity in Montserrat iguanas than in Saba iguanas. Montserrat iguanas show a genetic signature close to that of iguanas from French Guiana (Fig. 2). However, this did not appear to be the result of hybridization between two populations as several alleles are also present in the Central and South American clade populations (unpublished data). This should be investigated in a further study of the Montserrat population to check for the presence of exotic iguanas.

### Taxonomic remarks

According to Miralles et al. (2017), in their review of the taxonomic revision of *Amblyrhynchus cristatus*, there is no objective definition of subspecies, but for these authors, the subspecies rank is useful to refer to distinct population-level units that have not attained independent evolutionary lineage status, and so do not meet the species criteria. These authors thus described five new subspecies of *Amblyrhynchus cristatus* based on morphology, mitochondrial DNA, and microsatellite clusters as we did in this study. Most of their subspecies live on a single island, while two of them live on two islands and one island has two subspecies.

Breuil et al. (2019) discussed the arguments for naming the St. Lucia and Grenadines populations as one subspecies or a new species with two subspecies. The choice was made to adopt the most conservative solution for this emblematic species. The difficulty lies mainly in the different concepts used to distinguish between the species and subspecies categories (Torstrom et al. 2014). Hybridization is known between the endemic iguanas of the Lesser Antilles and the two invasive lineages i.e., *Iguana iguana* and *Iguana rhinolopha* (Breuil 2013, 2016; Vuillaume et al. 2015) and now with the Curacao lineage in Saint Maarten (Van den Burg et al. 2018a, 2018b). In addition, hybridization has recently occurred between endemic *Cyclura nubila* and *Iguana iguana* (Moss et al. 2018). In this context, it is clear that there are no intrinsic barriers to the reproduction among all the clades of *Iguana* identified first by Malone et al. (2000) and their subsequent work (Stephen et al. 2013). We chose to recognize these melanistic iguanas at the species level, integrating the source population of Venezuela and making it fit the criterion of an independent evolutionary unit.

For example, *Cyclura nubila nubila* differs by less than 1% from the ND4 sequence of *C. nubila caymanensis* . The same level of divergence is also found between *C. cychlura cychlura* and *C. cychlura inornata* which are considered valid subspecies (Malone and Davis 2004). With 1.47 % (12/818) difference between the two haplotypes (JQ340913, JQ340914) from Brazil and the two haplotypes from Saba-Montserrat (HM352505) and Venezuela (HM352501), the differences are greater than in these *Cyclura* subspecies. Powell and Henderson (2005), Powell et al. (2005), Powell (2006), and Henderson and Powell (2009) suggested that the Saba and Montserrat populations (and possibly the historical population on St. Croix) may warrant designation as a separate species from the Common Green Iguana found elsewhere. We follow their recommendations here.

According to Stephen et al. (2013), the Central American clade (Mexico to northern Panama) is distinct from the South American clade and suggests the presence of cryptic species. The difficulty with naming these two clades is that many authors, such as Lazell (1973) or Stephen et al. (2013) considered that the only morphological distinction between these two clades is “enlarged tubercle scales on the snout” (i.e., horns). Breuil (2013; 2016) demonstrated that there are many morphological differences between these clades and that, in addition, the horns of iguanas of the southern group of Lesser Antilles are different from the horns of *rhinolopha* from Central America. Even if some populations or some individuals in some Central American populations have only small or no horns at all, this does not change the taxonomic issue. The first Central American iguanas in Mexico were described as a full species, *Iguana rhinolopha*, which clearly belongs to the Central American clade. This name should therefore be used for the Central American clade from Mexico to northern Panama, called number III by Stephen et al. (2013) (Fig. 3).

Clade II of (Stephen et al. 2013) comprises subclade IIa, called north west of Andes, which contains iguanas from Columbia, Peru and Ecuador, whereas subclade IIb, called south east of Andes, includes iguanas from Venezuela, Suriname, Brazil, and the Lesser Antilles (Saba, Montserrat, St. Lucia). According to our results, in our working region, we have the choice to consider that we have one species (*Iguana iguana*) with three subspecies (*iguana*, *sanctaluciae*, *insularis*), and *Iguana melanoderma* as another species, with the following distribution:

• *Iguana iguana iguana* (French Guiana and Brazil);

• *Iguana iguana sanctaluciae* (St. Lucia);

• *Iguana iguana insularis* (St. Vincent and Grenadines);

• *Iguana melanoderma* (north-eastern Venezuela, Venezuelan coastal islands, Saba-Montserrat Puerto Rico Bank, Virgin Islands, St. Croix Bank).

According to Stephen et al. (2013), populations in Brazil, Suriname and Venezuela have the same unique PAC haplotype (JN811116) as the Saba-Montserrat iguanas (23 iguanas), which is different from the haplotypes found in clades IIb and III. The single PAC haplotype identified in Saint Lucia (also present in the Grenadines populations, unpublished results) is a synapomorphy of the iguanas of this southern group. So, we have now recognized one new species, *Iguana insularis*, with two subspecies:

• *Iguana insularis insularis* comb. nov. of the Grenada Bank (including the Grenadines);

• and *Iguana insularis sanctaluciae* comb. nov. from Saint Lucia.

Naming species and subspecies in the South American clade has not resolved phylogenetic relationships with continental iguanas. For example, the formation of the Isthmus of Panama which was previously thought to be 2.5 My old (Iturralde-Vinent 2006) may be more than 10 My older (Montes et al. 2015). This new dating challenges the age of the first colonization of Central America by ancestral iguanas. Our taxonomic proposal simply reflects the morphological and genetic originalities of the endemic insular horned iguanas of the southern Antilles and the melanistic iguanas of the northern Antilles, regardless of changes in the original natural distribution area. The characterization of all these insular endemic taxa provides a solid basis for conservation.

### Conservation implications

The description of *Iguana melanoderma*, with its morphological and genetic diagnostic characteristics, will enable conservationists to differentiate between endemic and exotic iguanas. For example, the IUCN Red List (Bock et al. 2018) considered the Common Green Iguana, *Iguana iguana*, to be of “Least Concern” but failed to differentiate between populations that do not have the same threat levels. With our taxonomic proposal, these endemic insular populations will be considered as a conservation unit with their own assessments.

As the range of invasive iguanas increases worldwide (Falcón et al. 2013), and mainly in the Caribbean (Powell et al. 2011; van den Burg et al. 2018a, 2018b), the probability of invasive iguanas arriving on Saba increases with trade, travel, and tourism on St. Maarten (Yokoyama 2012), another Dutch island 45 km north of Saba. It is thus necessary to conserve these original populations and to be able to differentiate them from invasive iguanas of different lineages.

The Saba Conservation Foundation (SCF) is responsible for nature management on the island for the local government and runs programs for the Saban Black Iguana by reducing defoliation by goats through the restriction of the number of free-ranging domestic animals (Powell et al. 2005). As a seed dispersing animal, this taxon contributes to the recolonization of landscapes after human, animal or natural destruction.

We found no evidence of genetic introgression in the Saban Black Iguana population; the samples studied for genetic analysis, collected in 2011, have all the characteristics of this new taxon. However, according to a photograph published in the book by Powell et al. (2005) (fig. 92), but not taken by these authors, invasive iguanas could be (or could have been) present in Saba, unless the locality of the photograph is in error.

Moreover, it is clear from the Google photographs of iguanas taken in the Virgin Islands that the invasive iguana (*Iguana rhinolopha*) is also present and hybridization is occurring as shown by the presence of intermediate phenotypes in these islands and by genetic data (Falcón et al. 2018). The same holds true for Margarita Island (Venezuela).

*Iguana iguana* is listed in CITES Appendix II, but export quotas exist for many countries for pet trade and products (leather, meat). No distinction is made between native and introduced populations, or between continental and insular populations (Powell and Henderson 2007). With regard to its endemicity [1], the small size of these islands (Saba: 13 km^2^, Montserrat: 100 km^2^) [2], that this new taxon occupies less than half of Saba and occupied less than 10 % of Montserrat before the eruption of the Soufrière Hills in 1995 [3], that the total effective population of this species in Saba and Montserrat could be around 400 adults [4], that there is a pressure on the biotope [5], that hunting is rare but present [6], that dogs and cats, as well as motor vehicles, kill iguanas [7], and that the invasive iguanas could arrive soon [8], this new taxon is, based on IUCN criteria, critically endangered on the islands of Saba and Montserrat.

Priority actions for the conservation of the species *Iguana melanoderma* are biosecurity [1], minimization of hunting [2], and habitat conservation [3]. The maritime and airport authorities of both islands must be vigilant about the movements of iguanas, or their sub-products, in either direction, even if the animals remain within the same nation’s territory. Capacity-building and awareness-raising should strengthen the islands’ biosecurity system and could enhance pride in this flagship species.

Key stakeholders in conservation efforts are the Dutch Caribbean Nature Alliance (DCNA), the Saba Conservation Foundation (SCF), the Montserrat National Trust (MNT) and the UK Overseas Territories Conservation Forum (UKOTCF).

The DCNA is already involved in the conservation of *Iguana delicatissima* on St Eustatius^1^. The MNT is implementing a project funded by the Darwin Initiative entitled “Adopt a Home for Wildlife”, which can also be harnessed for iguana conservation. MNT and UKOTCF suggested the following priority actions for the native iguana: preparation of an inventory, including a survey of the geographical distribution and ecology of the taxon, assessment of conservation status and regular monitoring, as well as habitat mapping and assessment of conservation purposes. The listing of Fox’s Bay Bird Sanctuary as a Ramsar Site was also recommended^2^.

In conclusion, the pioneering work of Malone et al. (2000) and her subsequent works (Malone and Davis 2004; Stephen et al. 2013), followed by those of Breuil (2013, 2016), Vuillaume et al. (2015), and Breuil et al. (2019) clearly demonstrated that there are morphological, mitochondrial, and nuclear divergences between what was considered to be a single species, *Iguana iguana*. Thus, different clades have been identified genetically and morphologically without naming them. These diagnosable insular entities are here considered to form two new species: *Iguana melanoderma* and *Iguana insularis*. Southeastern Andes populations are considered to be *Iguana iguana* and populations in the northern Isthmus of Panama are considered to be *Iguana rhinolopha*. Morphological, genetic, ecological, and ethological studies are needed to characterize and name all lineages not included in these four recognized species. Our work provides the tools for a better protection of these insular flagship species, mainly against alien iguanas.

## Supplementary Material

XML Treatment for Iguana melanoderma

## Footnotes

http://www.dcnanature.org/lesser-antillean-iguana/

http://www.ukotcf.org/CP/monserrat.htm

## References

[B1] Bandelt HJ Forster P Rohl A (1999) Median-joining networks for inferring intraspecific phylogenies. Molecular Biology and Evolution 16: 37–48. 10.1093/oxfordjournals.molbev.a026036 10331250

[B2] Barbour T (1937) Third list of Antillean reptiles and amphibians. Bulletin of the Museum of Comparative Zoology 82: 77–166.

[B3] Blankenship JR (1990) The Wildlife of Montserrat including an annotated bird list for the island. Montserrat National Trust, Montserrat, West Indies.

[B4] Bochaton C Grouard S Cornette R Ineich I Lenoble A Tresset A Bailon S (2015) Fossil and subfossil herpetofauna from Cadet 2 Cave (Marie-Galante, Guadeloupe Islands, F. W. I.): Evolution of an insular herpetofauna since the Late Pleistocene. Comptes Rendus Palevol 14: 101–110. 10.1016/j.crpv.2014.10.005

[B5] Bock B Malone CL Knapp C Aparicio J Avila-Pires TCS Cacciali P Caicedo JR Chaves G Cisneros-Heredia DF Gutiérrez-Cárdenas P Lamar W Moravec J Perez P Porras LW Rivas G Scott N Solórzano A Sunyer J (2018) *Iguana iguana*. The IUCN Red List of Threatened Species 2018: e.T174481A1414646.

[B6] Breuil M (2002) Histoire naturelle des Amphibiens et Reptiles terrestres de l’Archipel Guadeloupéen. Guadeloupe, Saint-Martin, Saint-Barthélemy. Patrimoines naturels, 54. Institut d’Écologie et de Gestion de la Biodiversité, Muséum National d’Histoire Naturelle, Paris, 339 pp.

[B7] Breuil M (2009) The terrestrial herpetofauna of Martinique: Past, present, future. Applied Herpetology 6: 123–149. 10.1163/157075408X386114

[B8] Breuil M (2013) Caractérisation morphologique de l’iguane commun *Iguana iguana* (Linnaeus, 1758), de l’iguane des Petites Antilles *Iguana delicatissima* Laurenti, 1768 et de leurs hybrides. Bulletin de la Société Herpétologique de France 147: 309–346.

[B9] Breuil M (2016) Morphological characterization of the common iguana*Iguana iguana* (Linnaeus, 1758), of the Lesser Antillean Iguana*Iguana delicatissima* Laurenti, 1768 and of their hybrids. Same paper as Breuil (2013) translated into English by International Reptiles Conservation Foundation (IRCF).

[B10] Breuil M Guiougou F Questel K Ibéné B (2010) Modifications du peuplement herpétologique dans les Antilles françaises. Disparitions et espèces allochtones. 2e partie: Reptiles. Le Courrier de la Nature 251: 36–43.

[B11] Breuil M Vuillaume B Schikorski D Krauss U Morton M Haynes P Daltry J Gaymes G Gaymes J Bech N Jelić M Grandjean F (2019) A story of nasal horns: two new subspecies of *Iguana* Laurenti, 1768 (Squamata, Iguanidae) in Saint Lucia, St Vincent and the Grenadines, and Grenada (southern Lesser Antilles). Zootaxa 4608: 201–232. 10.11646/zootaxa.4608.2.1 31717144

[B12] Daltry JC (1995) Report on the Findings of the Amphibians and Reptiles Team Part A: Species and Habitat Associations. Montserrat National Trust and Division of Forestry and Environment. Montserrat Biodiversity Project, Fauna and Flora International, Montserrat.

[B13] Darriba D Taboada GL Doallo R Posada D (2012) jModelTest 2: more models, new heuristics and parallel computing. Nature Methods 9: 772–772. 10.1038/nmeth.2109 PMC4594756 22847109

[B14] Dubois A (2009) Endangered species and endangered knowledge. Zootaxa 2201: 26–29. 10.11646/zootaxa.2201.1.5

[B15] Dunn ER (1934) Notes on *Iguana*. Copeia 1934: 1–4. 10.2307/1436422

[B16] Earl DA Vonholdt BM (2012) STRUCTURE HARVESTER: a website and program for visualizing STRUCTURE output and implementing the Evanno method. Conservation Genetics Resources 4: 359–361. 10.1007/s12686-011-9548-7

[B17] Edgar P (2009) The Amphibians and Reptiles of the UK Overseas Territories. Crown Dependencies and Sovereign Base Areas. Species Inventory and Overview of Conservation and Research Priorities. The Herpetological Conservation Trust, Bornemouth, UK.

[B18] Evanno G Regnaut S Goudet J (2005) Detecting the number of clusters of individuals using the software STRUCTURE: a simulation study. Molecular Ecology 14: 2611–2620. 10.1111/j.1365-294X.2005.02553.x 15969739

[B19] Falcón W Ackerman JD Recart W Daehler CC (2013) Biology and Impacts of Pacific Island Invasive Species. 10. *Iguana iguana*, the Green Iguana (Squamata: Iguanidae). Pacific Science 67: 157–186. 10.2984/67.2.2

[B20] Falcón W De Jesus Villanueva C Van den Burg MP Malone CL (2018) Disentangling the Origin of Common Green Iguanas in the Virgin Islands. IUCN SSC Iguana Specialist Group Annual Meeting, Forth Worth Zoo, Texas.

[B21] Garman S (1887) On West Indian reptiles in the Museum of Comparative Zoology, at Cambridge, Massachussetts. Proceedings of the American Philosophical Society 24: 278–286.

[B22] Gentile G Snell H (2009) *Conolophus marthae* sp. nov. (Squamata, Iguanidae), a new species of land iguana from the Galapagos Archipelago. Zootaxa 2201: 1–10. 10.11646/zootaxa.2201.1.1

[B23] Goudet J (2001) FSTAT, a program to estimate and test gene diversities and fixation indices.

[B24] Grouard S (2001) Subsistance, systèmes techniques et gestion territoriale en milieu insulaire antillais précolombien. Exploitation des Vertébrés et Crustacés aux époques Saladoïdes et Troumassoïdes de Guadeloupe (400 av. J.-C. à 1500 ap. J.-C.). PhD thesis, Muséum national d’Histoire naturelle, Paris.

[B25] Hamilton M Clubbe C Robbins SK Bárrios S (2008) Plants and habitats of the Centre Hills and Montserrat. In: Young RP (Ed.) A biodiversity assessment of the Centre Hills, Montserrat.Durrell Conservation Monograph N°1 Jersey, Channel Islands, 40–55.

[B26] Harford CL Pringle MS Sparks RSJ Young SR (2002) The Volcanic evolution of Montserrat using 40Ar/39Ar geochronology. In: Druitt TH Kokeelar BP (Eds) The Eruption of Soufrière Hills Volcano, Montserrat, from 1995 to 1999.The Geological Society of London, London, 93–113. 10.1144/GSL.MEM.2002.021.01.05

[B27] Hasegawa M Kishino H Yano TA (1985) Dating of the human ape splitting by a molecular clock of mitochondrial DNA. Journal of Molecular Evolution 22: 160–174. 10.1007/BF02101694 3934395

[B28] Hedges SB Heinicke MP (2007) Molecular phylogeny and biogeography of west Indian frogs of the genus *Leptodactylus* (Anura, Leptodactylidae). Molecular Phylogenetics and Evolution 44: 308–314. 10.1016/j.ympev.2006.11.011 17196836

[B29] Henderson RW Powell R (2009) Natural History of West Indian Reptiles and Amphibians. University Press of Florida, Miami, 496 pp.

[B30] Hofman CL Hoogland MLP (1991) The later prehistory of Saba, N.A.: the settlement site of Kelbey’s Ridge (1300–1450 A.D.). In: Ayubi EN, Haviser JB (Eds) Proceedings of the 13^th^ International Congress for Caribbean Archaeology Anthropological Institute of the Netherlands Antilles Archaeology, Anthropological Institute of the Netherlands Antilles, 477–492.

[B31] Hofman CL Hoogland MLP (2003) Plum Piece Evidence for Archaic Seasonal Occupation on Saba, Northern Lesser Antilles around 3300 BP. Journal of Caribbean Archeology 4: 12–27.

[B32] Iturralde-Vinent MA (2006) Meso-Cenozoic Caribbean paleogeography: implications for the historical biogeography of the region. International Geology Review 48: 791–827. 10.2747/0020-6814.48.9.791

[B33] Katoh K Kuma K Toh H Miyata T (2005) MAFFT version 5: improvement in accuracy of multiple sequence alignment. Nucleic Acids Research 33: 511–518. 10.1093/nar/gki198 15661851 PMC548345

[B34] Kumar S Stecher G Tamura K (2016) MEGA7: Molecular Evolutionary Genetics Analysis Version 7.0 for Bigger Datasets. Molecular Biology and Evolution 33: 1870–1874. 10.1093/molbev/msw054 27004904 PMC8210823

[B35] Lazell JD (1973) The Lizard Genus *Iguana* in the Lesser Antilles. Bulletin of the Museum of Comparative Zoology 145: 1–28.

[B36] Leigh JW Bryant D (2015) POPART: full-feature software for haplotype network construction. Methods in Ecology and Evolution 6: 1110–1116. 10.1111/2041-210X.12410

[B37] Leighton GRM Hugo PS Roulin A Amar A (2016) Just Google it: assessing the use of Google Images to describe geographical variation in visible traits of organisms. Methods in Ecology and Evolution 7: 1060–1070. 10.1111/2041-210X.12562

[B38] Lescure J (2000) Répartition passée de *Leptodactylus fallax* Müller, 1923 et d’*Eleutherodactylus johnstonei* Barbour, 1914 (Anoures, Leptodactylidés). Bulletin Société herpétologique de France 94: 13–23.

[B39] MacLean WP (1982) Reptiles and Amphibians of the Virgin Islands. MacMillan Caribbean, London, 54 pp.

[B40] Malone CL Davis SK (2004) Genetic contributions to Caribbean iguana conservation. In: Alberts AC Carter RL Hayes WK Martins EP (Eds) Iguanas: Biology and Conservation.University of California Press, Los Angeles, 45–57. 10.1525/california/9780520238541.003.0004

[B41] Malone CL Wheeler T Taylor JF Davis SK (2000) Phylogeography of the Caribbean rock iguana (*Cyclura*): Implications for conservation and insights on the biogeographic history of the West Indies. Molecular Phylogenetics and Evolution 17: 269–279. 10.1006/mpev.2000.0836 11083940

[B42] Malone CL Reynoso VH Buckley L (2017) Never judge an iguana by its spines: Systematics of the Yucatan spiny tailed iguana, *Ctenosaura defensor* (Cope, 1866) Molecular Phylogenetics and Evolution 115: 27–39. 10.1016/j.ympev.2017.07.010 28716742

[B43] Mayer GC (2012) Puerto Rico and the Virgin Islands. In: Powell R Henderson RW (Eds) Island Lists of West Indian Amphibians and Reptiles.Bulletin of the Florida Museum of Natural History 51(2), 136–147.

[B44] Miralles A Macleod A Rodríguez A Ibáñez A Jiménez-Uzcategui G Galo Quezada G Vences M Steinfartz S (2017) Shedding light on the Imps of Darkness: an integrative taxonomic revision of the Galápagos marine iguanas (genus *Amblyrhynchus*). Zoological Journal of the Linnean Society 181: 678–710. 10.1093/zoolinnean/zlx007

[B45] Montes C Cardona A Jaramillo C Pardo A Silva JC Valencia V Ayala C Pérez-Angel LC Rodriguez-Parra LA Ramirez V Niño H (2015) Middle Miocene closure of the Central American Seaway. Science 346(6231): 226–229. 10.1126/science.aaa2815 25859042

[B46] Moss JB Welch ME Burton FJ Vallee MV Houlcroft EW Laaser T Gerber GP (2018) First evidence for crossbreeding between invasive *Iguana iguana* and the native rock iguana (Genus *Cyclura*) on Little Cayman Island. Biological Invasions 20: 817–823. 10.1007/s10530-017-1602-2

[B47] Nei M Kumar S (2000) Molecular Evolution and Phylogenetics. Oxford University Press, New York, 333 pp.

[B48] Platenberg RJ Boulon RHJ (2006) Conservation status of reptiles and amphibians in the US Virgin Islands. Applied Herpetology 3: 215–235. 10.1163/157075406778116159

[B49] Powell R (2006) Conservation of the herpetofauna on the Dutch Windward Islands: St. Eustatius, Saba, and St. Maarten. Applied Herpetology 3: 293–306. 10.1163/157075406778905090

[B50] Powell R Henderson RW (2005) Conservation status of Lesser Antillean reptiles. Iguana 12: 62–77.

[B51] Powell R Henderson RW (2007) The St. Vincent herpetofauna. Applied Herpetology 4: 295–312. 10.1163/157075407782424539

[B52] Powell R Henderson RW Farmer MC Breuil M Echternacht AC Van Buurt G Romagosa CM Perry G (2011) Introduced amphibians and reptiles in the greater Caribbean: Patterns and conservation implications. In: Hailey A Wilson BS Horrocks JA (Eds) Conservation of Caribbean Island Herpetofaunas vol.2. Brill, Leiden, 63–143. 10.1163/ej.9789004183957.i-228.38

[B53] Powell R Henderson RW Parmalee JSJ (2005) The Reptiles and Amphibians of the Dutch Caribbean St. Eustatius, Saba and St. Maarten. STENAPA, St Eustatius, 192 pp.

[B54] Pregill GK Steadmann DW Watters DR (1994) Late Quaternary vertebrate faunas of the Lesser Antilles : historical components of Caribbean biogeography. Bulletin of the Carnegie Museum of Natural History 30: 1–51.

[B55] Pritchard JK Stephens M Donnelly P (2000) Inference of population structure using multilocus genotype data. Genetics 155: 945–959. 10835412 10.1093/genetics/155.2.945 PMC1461096

[B56] Reitz EJ (1994) Archeology of Trants, Montserrat. Part 2. Vertebrate Fauna. Annals of the Carnegie Museum of Natural History 63(4): 297–317.

[B57] Rice WR (1989) Analyzing tables of statistical tests. Evolution 43: 223–225. 10.1111/j.1558-5646.1989.tb04220.x 28568501

[B58] Roobol MJ Smith AL (2004) Volcanology of Saba and St. Eustatius. Koninklike Nederlandse Akademie van Wetenschappen. Royal Netherlands Academy of Arts and Sciences, Amsterdam, The Netherlands, 320 pp.

[B59] Schmidt KP Inger RF (1957) Living Reptiles of the World. Hanover House, New York, 287 pp.

[B60] Steadman DW Waters DR Reitz EJ Pregill GK (1984) Vertebrates from archeological sites on Montserrat, West Indies. Annals of the Carnegie Museum of Natural History 53: 1–29.

[B61] Stephen CL Reynoso VH Collett WS Hasbun CR Breinholt JW (2013) Geographical structure and cryptic lineages within common green iguanas, *Iguana iguana*. Journal of Biogeography 40: 50–62. 10.1111/j.1365-2699.2012.02780.x

[B62] Torstrom SM Pangle KL Swanson BJ (2014) Shedding subspecies: The influence of genetics on reptile subspecies taxonomy. Molecular Phylogenetics and Evolution 76: 134–143. 10.1016/j.ympev.2014.03.011 24662681

[B63] Underwood G (1962) Reptiles of the Eastern Caribbean. Caribbean Affairs (N.S.) 1: 1–192.

[B64] Valette V Filipova L Vuillaume B Cherbonnel C Risterucci AM Delaunay C Breuil M Grandjean F (2013) Isolation and characterization of microsatellite loci from *Iguana delicatissima* (Reptilia: Iguanidae), new perspectives for investigation of hybridization events with *Iguana iguana*. Conservation Genetics Resources 5: 173–175. 10.1007/s12686-012-9761-z

[B65] Van Buurt G (2005) Field Guide to the Amphibians and Reptiles of Aruba, Curaçao and Bonaire. Chimaira, Frankfurt, 137 pp.

[B66] Van den Burg MP Breuil M Knapp CR (2018a) *Iguana delicatissima* The IUCN Red List of Threatened Species 2018: e.T10800A122936983. 10.2305/IUCN.UK.2018-1.RLTS.T10800A122936983.en

[B67] Van den Burg MP Meirmans PG van Wagensveld TP Kluskens B Madden H Welch ME Breeuwer JAJ (2018) The Lesser Antillean Iguana (*Iguana delicatissima*) on St. Eustatius: genetically depauperate and threatened by ongoing hybridization. Journal of Heredity 109: 426–437. 10.1093/jhered/esy008 29471487

[B68] Vuillaume B Valette V Lepais O Grandjean F Breuil M (2015) Genetic evidence of hybridization between the endangered native species *Iguana delicatissima* and the invasive *Iguana iguana* (Reptilia, Iguanidae) in the Lesser Antilles: management implications. PLoS ONE 10. 10.1371/journal.pone.0127575 PMC4457794 26046351

[B69] Weir BS Cockerham CC (1984) Estimating F-statistics for the analysis of populations structure Evolution 38: 1358–1370. 10.1111/j.1558-5646.1984.tb05657.x 28563791

[B70] Yokoyama M (2012) Reptiles and Amphibians Introduced on St Martin, Lesser Antilles. IRCF Reptiles and Amphibians 19: 271–279.

[B71] Young RP Hilton GM (2008) Background to the Centre Hills biodiversity assessment. In: Young RP (Ed.) A biodiversity assessment of the Centre Hills.Montserrat. Durrell Conservation Monograph N°1 Jersey, Channel Islands, 30–39.

